# Multifaceted B cell response to transient HIV viremia in elite controllers

**DOI:** 10.1371/journal.ppat.1013817

**Published:** 2026-01-16

**Authors:** Luke Muir, Ondrej Suchanek, Peter Thomas, Sarah A. Griffith, Emma Touizer, Chloe Rees-Spear, Robert Krause, Masahiro Yoshida, Christopher L. Pinder, Rutuja Patil, Marko Z. Nikolic, Katie J. Doores, Marit J. Van Gils, Alasdair Leslie, Alex Sigal, Ravindra K. Gupta, Menna R. Clatworthy, Laura E. McCoy

**Affiliations:** 1 Division of Infection and Immunity, Institute for Immunity and Transplantation, University College London, London, United Kingdom; 2 Molecular Immunity Unit, Department of Medicine, Medical Research Council Laboratory of Molecular Biology, University of Cambridge, Cambridge, United Kingdom; 3 Cambridge University Hospitals NHS Foundation Trust, and NIHR Cambridge Biomedical Research Centre, Cambridge, United Kingdom; 4 Africa Health Research Institute, Durban, South Africa; 5 UCL Respiratory, Division of Medicine, University College London, London, United Kingdom; 6 Department of Infectious Diseases, School of Immunology & Microbial Sciences, King’s College London, London, United Kingdom; 7 Department of Medical Microbiology and Infection Prevention, Amsterdam UMC, University of Amsterdam, Amsterdam, the Netherlands; 8 Infectious Diseases, Amsterdam Institute for Infection and Immunity, Amsterdam, the Netherlands; 9 Cambridge Institute of Therapeutic Immunology and Infectious Disease (CITIID), Cambridge, United Kingdom; 10 Department of Medicine, University of Cambridge, Cambridge, United Kingdom; 11 Cellular Genetics, Wellcome Sanger Institute, Cambridge, United Kingdom; Medical Research Council Laboratory of Molecular Biology, UNITED KINGDOM OF GREAT BRITAIN AND NORTHERN IRELAND

## Abstract

Chronic HIV infection drives B cell dysfunction associated with the accumulation of tissue-like memory (TLMs) and activated memory B cells (MBCs) but decline in resting memory B cells. TLMs express multiple inhibitory receptors and lack response to soluble antigens. However, their origin and the mechanisms driving their expansion in HIV infection remain unclear. From bulk heavy chain BCR sequencing of MBC subsets from 5 PLWH with no detectable viremia, we hypothesized that TLMs (CD21- CD27- B cells) were significantly less mutated but also less diverse than other MBCs, suggesting an enrichment for innate-like B cells or that they belong to a less mature subset. Subsequent detailed multi-omics study of an immune response to a transient HIV viremia in an elite controller demonstrated a functional increase in Env-reactive IgG and MBCs with non-TLM phenotype. Single-cell RNA/BCR sequencing of PBMCs enriched for B cells revealed an orchestrated TNF-α response followed by interferon-α and -γ responses across all B cell subsets. This study provides new insights into multifaceted functional B cell response to transient HIV viremia and highlights TLM heterogeneity.

## Author summary

HIV infection changes the proportions and types of immune cells in the blood. T cells are affected because they are directly infected by HIV. However, how HIV alters the traits of B cells, in particular a subset called tissue-like memory B cells, is not fully understood. These cells have been purported to arise due to excessive viral stimulation and be in an exhausted state unable to perform normal B cell functions. However, tissue-like memory B cells have also been found in uninfected controls post-vaccination and may be a normal stepping stone in the trajectory of a B cell towards becoming an antibody-secreting cell. In this study we investigated the antibody response and gene expression profiles of tissue-like memory B cells in an individual who had spontaneous control of HIV (in the absence of treatment) but experienced a transient episode of detectable virus in their blood. We saw no increase in tissue-like memory B cells in response to this viral episode and no enrichment for virus-specific tissue-like memory B cells. Our results suggested these cells were formed in an interferon-driven rather than antigen-driven process, and we identified two distinct subsets of tissue-like-memory B cells following distinct B cell pathways.

## Introduction

B cell dysfunction in people living with human immunodeficiency virus (HIV) (PLWH) has been widely described [1] and is associated with suboptimal B cell responses to both infection and vaccination [2–5]. Although antiretroviral therapy (ART) has proven effective in reducing the viral load in PLWH to undetectable levels and generally restores CD4^+^ T cell counts, B cell homeostasis is never fully restored even in those who have maintained ART and supressed viral loads for multiple years [6–8]. Some of the key changes associated with HIV-mediated B cell dysfunction include hypergammaglobulinemia and the dysregulation of certain B cell subsets, including increased numbers of immature transitional B cells, activated memory B cells, tissue-like memory B cells (TLMs) and plasmablasts, whilst resting memory B cell (MBC) numbers are typically reduced [1].

TLMs were first described in 2008 by Moir et al. and can account for >50% of the MBC population in untreated chronic HIV infection compared to ~5% in HIV-negative individuals [9]. Their name refers to their enriched expression of surface FcRL4, similar to the tissue B cell population previously identified in human tonsils [10]. TLMs lack expression of the typical MBC markers CD21 and CD27 and are CD10^-^ CD19^Hi^ CD20^Hi^ [9,11]. They have also been shown to be enriched for expression of Th1-associated transcription factor T-bet and integrin CD11c [9,12]. However, the most defining feature of this subset is their high expression of a variety of inhibitory receptors (alongside FcRL4), including CD22, CD85j and CD72 [9,12]. Further, TLMs typically display a chemokine receptor profile suggestive of homing to sites of inflammation, with increased expression of CXCR3 and CCR6 [9,12]. Functional analysis of TLMs initially indicated that they were significantly less responsive to soluble stimuli and appeared to have undergone fewer rounds of proliferation *in vivo* compared to classical MBCs, resulting in the hypothesis that these were an exhausted B cell subset driven by chronic antigen exposure during infection [9]. This hypothesis was further supported by the evidence that a knockdown of inhibitory receptor expression (including FCRL4, SIGLEC6 and CD85J) on these subsets using siRNA could partially restore the ability of TLMs to respond to soluble stimuli [13]. However, recent data has suggested that TLMs can respond to complex antigens, suggesting they may not be as functionally inert as first thought [14].

Expanded populations analogous to TLMs have been described in various other chronic inflammatory settings, such as Hepatitis B infection, Malaria infection, and systemic lupus erythematosus (SLE) [15–17] and acute infections such as SARS-CoV-2 [18,19]. Although a different label is often used for this B cell subset in each of these conditions (e.g., “atypical” B cells in malaria or “double negative” B cells in SLE), the cells appear to share several hallmark characteristics, including the expression of inhibitory receptors, T-bet and a CD21^lo^ MBC phenotype. Malaria-associated atypical MBCs are amongst the most studied; they expand during chronic infection forming up to 51% of total mature circulating B cells. Extensive scRNA sequencing analysis on TLM, atypical and DN cells from HIV, Malaria and SLE respectively, revealed remarkably similar transcriptional profiles for these populations [20]. Overall, this suggests that there is a potential common mechanism behind the induction and expansion of these cells during chronic inflammation arising from different disease states.

To date the mechanisms driving the development of TLM/atypical B cells have not yet been fully defined. Recent data from murine models has shown that the interferon (IFN)-γ dynamics associated with chronic LCMV infection can induce an epigenetically distinct B cell subset [21] whilst data from individuals who acquired malaria suggested an important role for IFN-γ [20,22,23]. In contrast, a previous study in PLWH with uncontrolled viremia, found the majority of HIV envelope glycoprotein (Env)-reactive MBCs were T-bet^+^, a marker which has been linked to TLMs, suggesting that there may be a role for antigen-driven effects [9,12,15,24]. However, as TLMs can account for ~50% of the class-switched MBC population during chronic HIV infection while antigen-specific account for <1%, we argue it is likely that generic inflammatory stimuli are involved in the induction of TLMs irrespective of their antigen specificity.

In this study, we explored the mechanisms underlying B cell dysfunction in HIV infection, focusing on TLM B cells, which are associated with inhibitory receptor expression and impaired antigen responsiveness. Using bulk B cell receptor (BCR) sequencing, we analyzed MBC subsets in elite HIV controllers and individuals taking ART. Our findings revealed that TLMs in HIV, characterized by a CD21^-^CD27^-^ phenotype, exhibit reduced mutational complexity and diversity compared to other MBCs, suggesting an enrichment of innate-like or less mature cells within this population. A multi-omics investigation of an elite controller during an episode of transient HIV viremia, which models early viral outgrowth uncovered dynamic immune responses. These included the emergence of non-TLM Env-reactive MBCs, IgG targeting the HIV Env protein, and activation signatures across B cell subsets. Single-cell analyses highlighted TLM heterogeneity, identifying two distinct subsets (TLM1 and TLM2) with varying differentiation states, IGHV4–34 usage, and mutational burdens. Additionally, pseudotime analyses suggested that TLMs comprise both innate-like and conventional MBCs, underscoring their complexity. These findings provide novel insights into B cell responses during HIV infection, with potential implications for vaccine and therapeutic strategies.

## Results

### TLMs are significantly less diverse and mutated than other MBC subsets in PLWH

**Fig 1:**
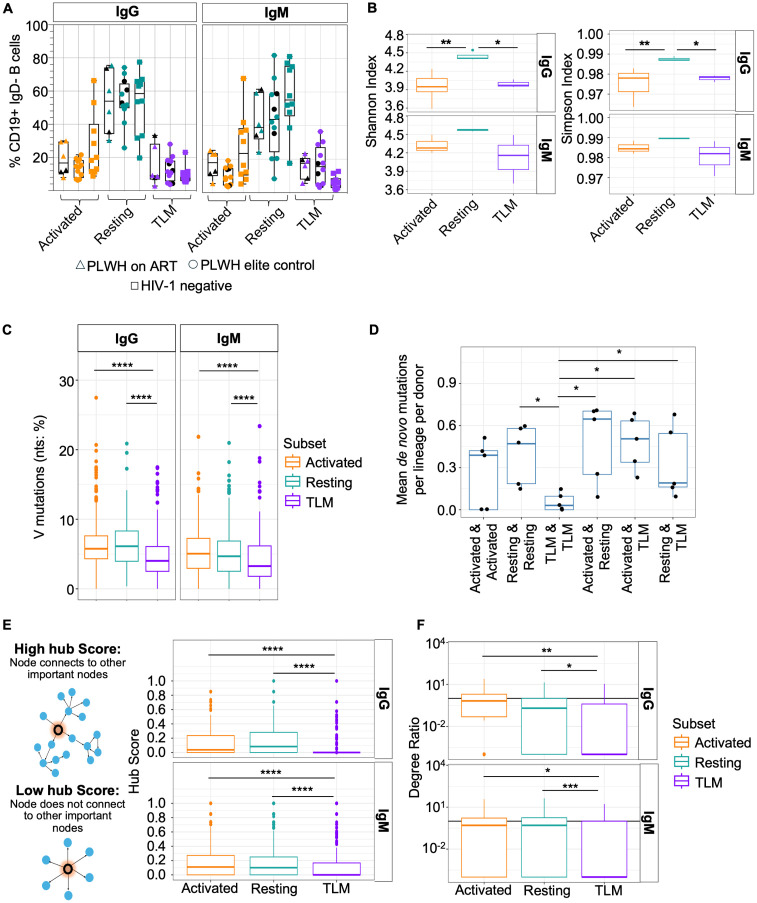
TLMs are significantly less diverse and mutated than other MBCs in PLWH. **A:** Flow-cytometric quantification of frequencies of IgM and IgG MBC subsets within IgD- B cells, n = 10 PLWH on ART (triangles), n = 12 for PLWH with elite control (circles) and n = 10 for HIV-negative controls (squares). Gating strategy shown in S1 Fig. MBC subsets are colour-coded as follows: activated (orange), resting (teal) and TLM (purple). Donors that underwent bulk BCR sequencing are highlighted with black symbols. No statistical significance was found in the Benjamini-Hochberg multiple testing adjusted p-values between donor groups for each MBC subset in turn using Mann-Whitney U tests with the compare_means function from the ggpubr package. **B:** Clonal diversity indices of MBC subsets stratified by IgG versus IgM expression for PLWH (n = 5). 100 BCR sequences were randomly sampled from each donor/subset for comparison. **C:** VH mutational burden (as % of nucleotides) of BCR sequences analysed in (**B)**. **D:** Calculation of mutations between B cell subsets for PLWH (n = 5). Lineages containing multiple MBC subsets were selected, and BCR sequences underwent pairwise comparison to identify unique AA mutations (FWR1 - FWR3). The median was taken per comparison for each lineage and then averaged to give a value per donor. The x axis shows the comparison group, and the y axis the average proportion of unique AA mutations per comparison. **E-F:** BCR phylogenetic network analysis to ascertain MBC subset evolution within PLWH (n = 5). Networks were created for each lineage, with each node representing a BCR (self-linkages excluded). Edges between nodes were drawn if the tip-to-tip distance was below a set threshold, defined as the position of first apex in the rate of change curve for the tip-to-tip distances. The network was directed by only allowing edges from closer nodes to germline to further nodes from germline. Once these networks were created, the hub score (**E**) and out-degree to in-degree ratio (**F**) was calculated (hub score representation, panel **(E)**, left hand side), to provide global and local measures of node connectivity, respectively. Throughout, statistical significance was assessed using Mann-Whitney U tests with the compare_means function from the ggpubr package (p > 0.05 not marked, p ≤ 0.05 *, p < 0.01, **, p < 0.001 ***, p < 0.0001 ****).

We first sought to understand if TLMs may be blocked in their ability to undergo antigen-driven evolution of their BCRs. We selected a variety of PLWH (with or without concurrent ART), including those in an elite control cohort, and phenotyped their circulating B cells by flow cytometry (Figs 1A and S1). There was inter-donor variation irrespective of whether the donors were in the ART or the elite control group and regardless of treatment status. The majority of MBCs were consistently of a resting phenotype and there was no obvious enrichment for a particular MBC subset within either group as compared to HIV negative controls. As no statistical significance was found between donor groups for each MBC subset in turn (Fig 1A) we randomly selected five donors (two from the ART control group and three from the elite control group) and bulk-sorted TLM (CD21^-^CD27^-^), resting (CD21^+^CD27^+^) and activated (CD21^-^CD27^+^) MBCs based on their isotype (IgG versus IgM). These cells then underwent bulk BCR sequencing of the IgG and IgM heavy chains (methods) to assess affinity maturation, diversity and evolution since these are key indicators of antigen-driven evolution in B cells. The aim of this was to compare BCR repertoire characteristics in different MBC populations during HIV infection. We observed that resting IgG MBCs were significantly (Mann-Whitney U test) more diverse than the other subsets, and that the diversity of IgG TLM and activated MBCs was similar (Fig 1B). A similar trend was observed for IgM MBCs (Fig 1B); however, this was not found to be statistically significant (p > 0.05) and may reflect their wider responsiveness to a range of antigens and distinct temporal germinal centre outputs.

Next, we sought to ascertain the mutational burden of these BCR sequences across MBC subsets. Firstly, the V_H_ mutation percentage was calculated (Fig 1C). In accordance with previous reports in HIV [25], TLMs were significantly less mutated than the other MBC subsets (p = 4.86x10^-20^, p = 4.45x10^-20^, p = 8.16x10^-9^, p = 2.76x10^-4^ for IgG activated vs TLM, IgG resting vs TLM, IgM activated vs TLM, and IgM resting vs TLM respectively) (pairwise Mann-Whitney U test). This may suggest that TLMs are less able to undergo antigen-driven activation and BCR mutation. To further characterize the mutation landscape of these cells, the proportion of unique (*de novo*) amino acid (AA) mutations between and within each subset was compared. The median unique mutation frequency was calculated per MBC subset for each lineage, and the mean average was taken per donor (Fig 1D). This data illustrates that mutation sharing is significantly greater between TLMs than between other subsets and suggests that they are less able to undergo continued BCR diversification than other MBC subsets since they contain fewer unique mutations overall.

Next, directed phylogenetic networks were created to better understand the evolutionary trajectories of the different MBC subsets. These networks allow evaluation of evolutionary importance of each node (cell) using the hub score (Fig 1E) and the out-degree to in-degree ratio (degree ratio; Fig 1F). The hub score estimates the value of each node’s connections to other nodes within the network, i.e., the score is higher if a cell connects to another cell that is highly connected. We observed significantly lower hub scores for TLMs than other subsets, regardless of isotype (IgG; Activated p = 5.5x10^-7^; Resting p = 7.1x10^-5^, IgM; Activated p = 2.3x10^-7^, Resting p = 4.4x10^-6^,.Fig 1E) (Mann-Whitney U test). This illustrates that MBCs are less able to undergo continued BCR evolution upon differentiation into TLMs than other subsets. Similarly, the degree ratios (ratio of outgoing connections divided by incoming connections) were significantly lower for TLMs across both isotypes than the other subsets (Fig 1F, IgG; Activated p = 0.0011; Resting p = 0.0308, IgM; Activated p = 0.0147, Resting p = 0.0005) (Mann-Whitney U test). Taken together, TLMs studied here contained significantly fewer mutations, were less diverse, and were less well connected in each B cell network than the other two MBC subsets that underwent heavy chain BCR sequencing from these 5 PLWH. Such data suggests that TLMs could be subject to additional activation/maturation controls as reported previously [14], a precursor population to other subsets, or produced via extrafollicular responses that undergo weaker affinity maturation [26].

### Minor transient spike in HIV viremia triggers a potent antibody response with expanded neutralization breadth

**Fig 2:**
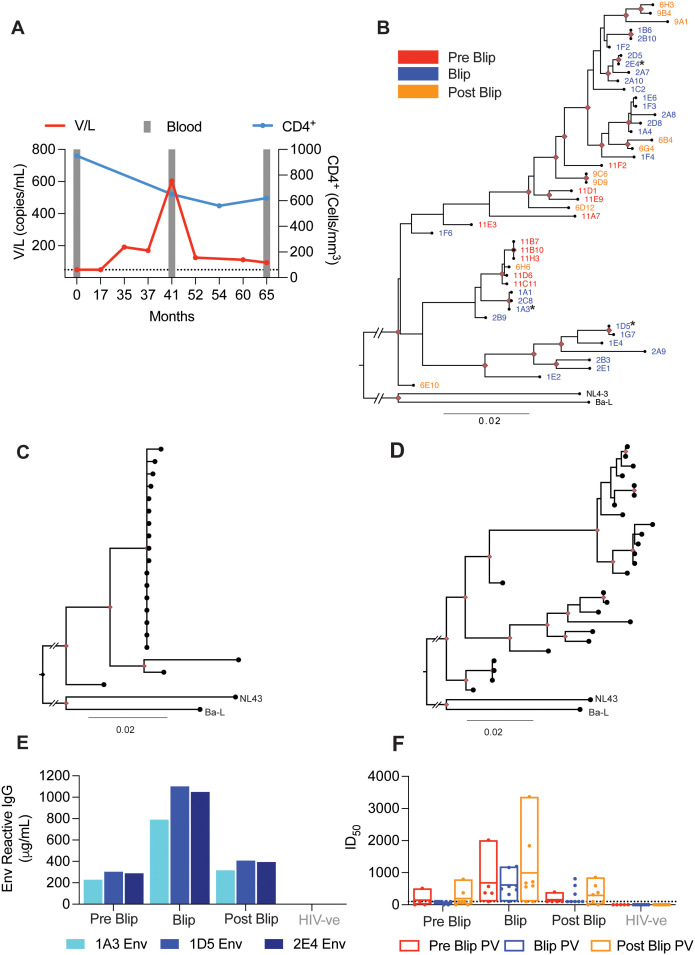
Minor transient spike in HIV viremia triggers antibody response. **A:** Dynamics of viral load (V/L, copies/mL) and CD4 + T cell count (per mm3) from an elite controller (index participant). Grey bars indicate timepoints of stored blood (PBMC/plasma) samples. **B:** Maximum Likelihood Phylogenetic analysis of env nucleotide sequences (amplicons spanning AA35-683, HXB2 numbering) isolated from the index participant. Sequences are coloured according to the sampling timepoint. Red nodes represent bootstrap support > 70%. Clade B sequences (NL4-3 and Ba-L) were included as outgroups for phylogeny rooting. Root branches have been shortened to prevent large branches caused by differences between participant sequences and outgroups. **C-D:** Maximum Likelihood Phylogenetic analysis of env nucleotide sequences (amplicons spanning AA35-683, HXB2 numbering) isolated from an individual on ART at one timepoint (**C)** or the index participant at the viral blip timepoint **(D)**. Red nodes represent bootstrap support > 70%. **E:** Anti-Env IgG titre in the plasma from the index and a HIV-negative participant. Plasma titre was assessed via semi-quantitative ELISA against the 1A3, 1D5 and 2E4 Env proteins (encoded by env sequences isolated from the index participant). **F:** Pseudovirus neutralization 50% inhibitory dilution (ID_50_) titres of plasma from the index and HIV-negative participant, respectively. Pseudovirus was expressed using autologous env sequences cloned from the index participant including 6 pseudoviruses from the pre blip timepoint (month 0), 8 pseudoviruses from the blip timepoint (month 41) and 8 pseudoviruses from the post blip timepoint (month 65). The dotted line represents the detection limit of the assay (ID_50_ = 1:100). If no neutralization was detected, samples were assigned an ID_50_ of 0. If only a single data point of the plasma dilution series was above 1:100, no accurate ID_50_ estimation could be determined and thus samples were assigned an ID_50_ of 1:100. The box plot lines show the median and 10-90th percentile.

Based on our BCR analysis of MBCs in PLWH, we hypothesised that the TLM subset may have impaired capacity for BCR mutation and diversification. To address how and when TLMs arise, we focussed further study on one donor who controlled virus in the absence of ART but then experienced a loss of control and a transient viral load, where longitudinal samples (pre-, during and post-viral blip) were available. Access to such timepoints allowed us to gain a unique insight into B cell dynamics during a relatively acute and low viral blip as may occur during the early stages following ART cessation. This ART naïve individual (referred to as “index participant”) had a low/undetectable viral load (<50 copies/mL) at the time of recruitment to the elite controller cohort (month 0 – Pre Blip) in October 2013. However, when sampled at month 41, a viral load of 603 copies/mL was detected (Blip). This viral blip was associated with a CD4^+^ T cell count decrease from 950 cells/μL to 650 cells/μL (Fig 2A). Following the viral blip, CD4^+^ T cell counts remained largely stable through to month 65, although they never fully recovered to the pre-blip levels. Similarly, the viral load returned to low levels of ~100 copies/mL at month 52 (Post Blip).

Analysis of the integrated HIV *envelope (env)* gene sequences by single genome amplification (SGA) from genomic DNA revealed a high degree of sequence variation within the index participant across pre-blip, blip and post-blip timepoints (Fig 2B). While certain branches of the phylogenetic tree were enriched with sequences from specific time points, the sequences from the post-blip time point were dispersed throughout the tree, suggesting limited selective pressure across the assessed timepoints despite ongoing replication. Interestingly, and in contrast to diverse *env* sequences from the viral blip (index participant) (Fig 2C), sequences recovered from an individual PLWH who was on ART (Fig 2D) were largely clonal, likely driven instead by the proliferation of infected T cell clones rather than viral outgrowth and diversification [27]. Moreover, the sequence diversity of the index participant viral quasi-species during the blip may have been underestimated as only integrated provirus was evaluated. This was because it was not possible to obtain viral sequences directly from replicating virus in the blood due to insufficient RNA recovery at this low viral load (<1,000 copies/mL).

To profile the B cell response to the viral blip we first determined the anti-Env plasma IgG titre. Env is the only viral antigen exposed on the surface of the HIV virion and is thus the major target for the B cell mediated antibody response, including neutralizing antibodies against HIV that are the goal of many HIV vaccine candidates. Three *env* sequences representative of the sequence variation and glycosylation features present in the index participant were selected, cloned and expressed as stabilized soluble recombinant proteins (1A3, 1D5 and 2E4) by utilizing SOSIP mutations [28]. The stabilized Env proteins were used to quantify the autologous anti-Env IgG titre and compared with the binding response against two non-autologous Env SOSIP proteins: BG505 N332 (Clade A) and B41 (Clade B) [28,29]. Strikingly, the anti-Env IgG response of the index participant rose to >1 mg/mL at the viral blip (Fig 2E). As expected, the titre against autologous Env proteins (Fig 2E) was higher than that against heterologous proteins and no response was observed in HIV-negative control plasma (S2 Fig). Interestingly, the IgG titre against the heterologous proteins peaked at the viral blip, suggesting that the small viral load increase was capable of boosting both the autologous and heterologous IgG titre.

Subsequently, plasma neutralization was assessed using a pseudovirus assay as previously described [30,31]. The 50% inhibitory dilution (ID_50_) values highlight that plasma neutralization was broadest and most potent against autologous pseudoviruses at the viral blip (Fig 2F), in line with the ELISA readings (Fig 2E). This is in contrast to weaker and less broad responses at the pre-blip and post-blip timepoints (Fig 2F). Interestingly, the viral blip timepoint plasma was unique in neutralizing a heterologous pseudovirus; B41, albeit at a low level (S2 Fig). Overall, the neutralization profile of the index participant appeared to be the weakest in terms of breadth and potency against autologous pseudoviruses prior to the viral blip (Fig 2F), followed by a ~ 5–10-fold increase in potency and increased breadth associated with the viral blip, before the response appears to partially return to baseline (Fig 2F).

Taken together, these data show that in an elite controller, even a minor spike in viral load (603 copies/mL) can induce a potent but short-lived neutralizing plasma antibody response against HIV. This makes the index participant a suitable case for studying B cell response to HIV viremia, including TLM development with particular relevance to the acute setting of ART cessation.

### Transient viral blip induces expansion of Env-reactive IgG^+^ memory B cells

**Fig 3:**
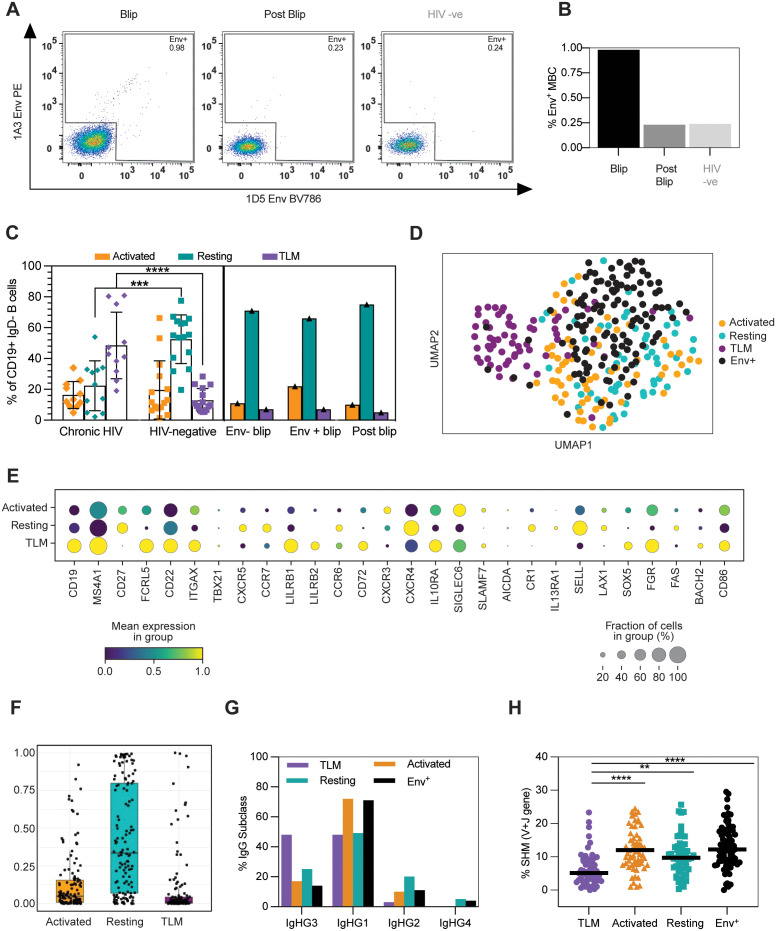
Transient viral blip induces expansion of Env-reactive IgG+ memory B cells. **A:** Gating strategy for FACS-sorting of 1A3 Env-PE and 1D5 Env-BV786 specific memory B cells (Aqua live/Dead-, CD4-, CD19 + , IgM- IgG+) from index participant at the blip (month 41) and post-blip (month 65) timepoints, or a HIV negative participant. **B:** Percentage of Env-reactive B cells within IgG + MBCs stratified by participant. **C:** Proportion of MBC subsets (resting, activated, TLM, intermediate) within total IgG + MBCs in PLWH with chronic viremia (n = 11) and HIV-negative (n = 15) controls are shown on the left panel. The analysis was based on flow-cytometric phenotyping of the expression of CD21 and CD27. Statistical significance was assessed using Mann-Whitney U tests for each subset in turn between the chronic viremia and HIV-negative donors (p > 0.05 not marked, p ≤ 0.05 *, p < 0.01, **, p < 0.001 ***, p < 0.0001 ****). The right panel shows data on these MBC subsets for the elite controller during the viral blip (divided into Env+ and Env- cells) compared to the post-blip timepoint. **D:** UMAP visualization of single cell transcriptomes from TLM, activated and resting MBCs (n = 301) sorted from the elite controller post blip and Env+ memory B cells from the blip timepoint. Single cell libraries were prepared via the Smart-seq 2 pipeline [32], a total of 88 resting, 88 activated, 88 TLM and 161 Env + MBC are profiled. Cells are annotated by their sorting identity. **E:** Expression of selected genes associated with memory B cell subsets profiled in **(D)**. The fraction of cells is shown by the dot size, and the mean expression level is reflected by the colour. **F:** Similarity of single-cell transcriptomes of Env + IgG+ memory B cells with the resting, activated and TLM IgG + B cell subsets sequenced in **(D)**. Similarity is calculated as probability using the Celltypist annotation tool trained on the sorted post-blip MBC subsets. **G-H:** IgG subclass usage (**G**) and nucleotide somatic hypermutation levels (V + J gene), (**H**)of sequenced memory B cell populations shown in (**D**). One way-ANOVA with Tukey’s multiple comparison used for statistical testing. *p < 0.032, **p < 0.021, ***p < 0.0002 and ****p < 0.0001.

After characterizing the humoral response to this minor viral blip, we next examined potential changes in the MBC compartment. We used fluorochrome-tagged autologous 1A3 and 1D5 recombinant Env proteins to detect antigen-specific MBCs at the blip and post-blip timepoints. Unfortunately, limited PBMC availability precluded such analysis in the pre-blip sample. Env^+^ IgG ^+^ MBCs formed almost 1% of MBCs at the blip timepoint (Fig 3A and 3B). However, this population contracted by 75% at the post-blip timepoint as the index participant regained viral control (Fig 3B), mirroring our ELISA and neutralization data (Fig 2E and 2F). Surprisingly, the flow-cytometric phenotyping of Env-negative and Env-positive IgG^+^ B cells during the blip (and all IgG^+^ MBC post-blip) demonstrated a marked similarity to the MBC phenotypes seen in 15 HIV-negative donors with the resting subset representing >75% and TLM < 10% of MBCs, respectively (Fig 3C). This contrasts with the MBC phenotypes of 11 donors with chronic viremia (Fig 3C) who had significantly expanded TLM and activated subsets and a reduced proportion of resting MBCs compared to HIV-negative controls (Fig 3C). Although there was no enrichment of TLM cells in the Env^+^ subset during the viral blip, as has been previously reported for PLWH with high chronic viremia [25], we did observe a > 2-fold enrichment in the frequency of the activated subset in the Env^+^ MBCs compared to the frequency of the activated subset in Env-negative MBCs [33].

Next, we used FACS to isolate IgG+ cells from all three memory subsets (resting, activated and TLM) as well as Env^+^ IgG^+^ MBCs for single-cell RNA/BCR sequencing to assess their transcriptomic similarity. UMAP projection of the different MBC transcriptomes presented TLMs as a distinct population, whereas there was a significant overlap between the resting and activated MBC subsets (Fig 3D). Interestingly, the Env^+^ cells largely clustered with the resting and activated subsets but not TLMs (Fig 3D), mirroring their flow-cytometry phenotype (Fig 3C). TLM population identity/annotation was confirmed by their expression of *bona fide* TLM marker genes such as FCRL5 and ITGAX and low expression of CD27 when compared to the resting and activated subsets (Fig 3E). We subsequently used the automated annotation tool Celltypist to quantify the transcriptional similarity of the Env^+^ cells to MBC subsets. This confirmed that the vast majority of Env^+^ cells were transcriptionally most similar to resting MBCs, with only a small proportion of cells resembling TLMs (Fig 3F). Finally, we compared the differences in the use of IgG subclasses and levels of SHM among MBCs. In line with our earlier data (Fig 1C), TLM cells have significantly lower levels of SHM than resting and activated MBCs (Fig 3G). TLM cells also displayed decreased usage of downstream IgG subclasses such as IgG2 and IgG4, suggesting a less mature or more innate-like phenotype (Fig 3H). Again, Env^+^ MBCs had a BCR profile (SHM, IgG subclasses) more similar to resting/activated MBCs (Fig 3G and 3H).

Overall, these data confirm that the viral blip was accompanied by an expansion of activated Env^+^ IgG MBCs. However, this discrete viral blip was not large enough to induce the global MBC dysfunction typically associated with chronic HIV viremia nor dysfunction within Env^+^ MBCs. Instead, the antigen-specific MBCs observed (presumably precursors of anti-Env antibody-secreting cells) resembled resting/activated MBCs both phenotypically and transcriptionally. Although TLM cells were present, their frequency was significantly lower than in chronically infected individuals and more in keeping with HIV-negative donors.

### Transient viral blip induces a complex response across both B and non-B cell immune subsets

**Fig 4:**
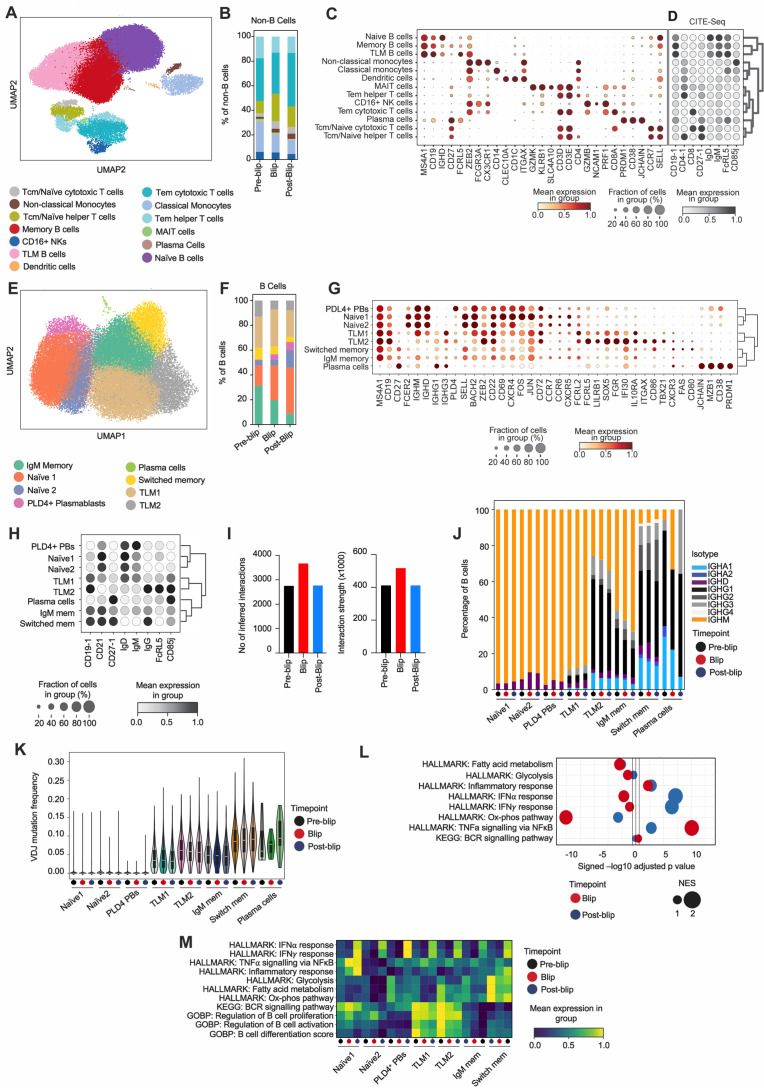
Transient viral blip induces a complex response across both B- and non-B cell subsets. **A:** UMAP visualization of annotated single-cell transcriptomes (48,602 cells) recovered from circulating MACS-selected B cells spiked with PBMC in 3:1 ratio of the index participant (elite controller) across all timepoints. **B:** Bar plot showing proportion (% of non-B cells) of non-B cell subsets across all three timepoints. **C-D:** Dot plot showing expression of selected marker genes **(C**) and CITE-seq protein markers (**D**) across annotated cell clusters from (**A)**. The fraction of cells expressing a particular marker is shown by dot size and the marker mean expression level is reflected by the colour. **E:** UMAP visualization of finely annotated B cell transcriptomes (42,149 cells) extracted from the dataset shown in **(A)**. **F:** Bar plot showing proportion (% of B cells) of annotated B cell subsets across all three timepoints. **G-H:** Dot plot showing expression of selected marker genes (**G**) and CITE-seq protein markers (**H**) across annotated B cell clusters from **(E)**. The fraction of cells expressing a particular marker is shown by dot size and the marker mean expression level is reflected by the color. **I:** Box plots showing number of inferred cell-cell interactions (left) and interaction strength (right) across timepoints using CellChat package. Both B- and non-B cells included. **J:** Isotype usage analysis depicted as % of B cells within each subset and timepoint. **K:** Violin plot showing VDJ mutation frequency stratified by subset and timepoint. **L:** GSEA of selected Hallmark and KEGG gene sets based on pre-ranked DEG comparing all B cells from blip (red dots) and post-blip timepoint (blue dots) to the reference (pre-blip B cells). Dot size reflects normalized enrichment score (NES). Vertical black lines indicate the threshold for statistical significance. **M:** Heatmap showing scanpy expression score of selected HALLMARK, KEGG and GOBP gene sets in each B cell subset and timepoints. Scaled by row.

To examine the transcriptomic changes in the whole B cell and non-B cell immune compartment of the index participant across all three time points in an unbiased approach, we performed single-cell RNA/BCR sequencing of MACS-selected circulating B cells spiked with all PBMCs at 3:1 ratio (favouring B cells). This generated a total of 48,602 single-cell transcriptomes (post-QC) with 13 distinct cellular clusters identified (Fig 4A and 4B). CellTypist automated cell annotation (Figs 4C, S3 and S4) [34] was validated and further refined using a panel of eight CITE-seq markers (S3 and S4 Figs).

The non-B cell compartment was analysed using differential abundance analysis (DA) with the milopy package across timepoints. Milopy uses the QL F-test with a specified contrast to compute a P value for each neighbourhood and controls for multiple testing as described previously [35]. The DA analysis revealed an expansion of naïve helper T cells during the viral blip (12% in blip to 24% post-blip; Figs 4D and S4). This was unexpected as increased viral load typically correlates with decreased helper T cells due to their direct destruction by HIV [36]. Indeed, the Tem/Effector helper T cells decreased during the viral blip by a greater magnitude (6% in blip to 2% post-blip). Interestingly, in the post-blip phase, we observed an expansion of effector memory (TEM)/ terminal effector memory (TEMRA) cytotoxic T cells (33.3% in blip to 46.8% post-blip, Figs 4B and S4) that likely played a dominant role in regaining viral control.

In the B cell compartment, we annotated eight distinct cell clusters (Fig 4E) based on their marker genes (Fig 4F) and CITE-seq panel expression (Fig 4G): Naive1, Naive2, PLD4 + plasmablasts, TLM1, TLM2, IgM memory, switched memory, and plasma cells. Notably, we identified two distinct TLM populations, both expressing canonical TLM markers although with a different intensity. TLM2 cells had higher expression of ITGAX, TBX21 (T-bet), LILRB1, and CD86 (Fig 4G). A similar expression pattern was also observed at the surface protein level by CITE-seq (Fig 4H). This suggests that TLM1 may be a precursor to the TLM2 population, which resembles previously described TLM populations more closely [9,20]. DA analysis with the milopy package was again used to examine the cell cluster changes in the B cell compartment across timepoints (S4 Fig). This analysis revealed a progressive increase in naïve B cells (20.4%, 32.6%, 51.1%) and that PLD4 + plasmablasts expand during the blip and are maintained post-blip (0.6%, 4.3%, 6.6%) (Fig 4H). The expansion of naïve B cells during and after the viral blip may result from their enhanced generation and/or recruitment to combat the transient viremia. In line with our previous flow cytometry and Smart-Seq2 data (Fig 3), the proportion of TLM cells remained stable during the viral blip.

The active coordination of antiviral immune response during the blip was further evidenced by the increased total number and strength of predicted cell-cell interactions accounting for all annotated immune cells (Figs 4I and S4). Of note, PLD4 + plasmablasts had one of the lowest numbers of predicted interactions at the blip timepoint, which may, together with predominant IGHM expression (Fig 4J) and lower levels of SMH (Fig 4K), indicate their extrafollicular differentiation pathway, allowing for a rapid anti-viral IgM response. Similarly, TLM1 cells predominantly expressed IGHM and had lower SHM burden compared to TLM2 that were mostly switched to IgG subclasses, indicating that TLM1 cells are less mature than TLM2 cells (Fig 4J and 4K). However, TLM2 were in turn less mature than switched memory as evidenced by more restricted SHM. During blip and post-blip, TLMs (unlike the switched memory subset) increased their proportion of unswitched cells with a small drop in median SHM, consistent with an influx of new (less mature) B cells in response to viremia (Fig 4J and 4K).

Analysis of Hallmark gene signatures for the whole B cell compartment across timepoints revealed a significantly enriched TNF-α signature during the blip, which declined post-blip when an enhanced interferon-α and -γ signature emerged instead (Fig 4L). This temporal signature pattern was seen across all B cell subsets, although with variable intensity (Fig 4M). The Naïve1 subset displayed the strongest TNF-α and inflammatory response during blip, while PLD4 + plasmablasts were the top expressor of both interferon signatures (Fig 4M). Unexpectedly, this global blip-induced interferon response was not accompanied by an expected TLM expansion [23], for which persistent viremia might be required. Surprisingly, TLM1 and TLM2 were the most active B cell subset in terms of BCR signalling, activation and differentiation with only slight decrease of these signatures during blip and post-blip phases (Fig 4M). This persistent TLM activation state might indicate evolving early dysfunction, ultimately leading to reduced responsiveness, as documented in TLMs from chronically viremic PLWH [9,14,37].

Taking together, our comprehensive unbiased single-cell data analysis elucidated dynamic changes in B cell and non-B cell compartments in response to a transient viral blip. This viral blip induced TNF-α and subsequent IFN response in all B cell subsets, with naïve B cells and newly emerged (likely extra-follicular) PLD4 + plasmablasts being the most robust responders. Surprisingly, TLM2 cells did not expand in response to the transient viremia but along with TLM1 cells displayed a persistently activated phenotype. Thus, this analysis emphasises the nuanced interplay between viral load and immune modulation in an elite HIV controller.

### TLM subsets are heterogenous and display innate-like B cell characteristics

**Fig 5:**
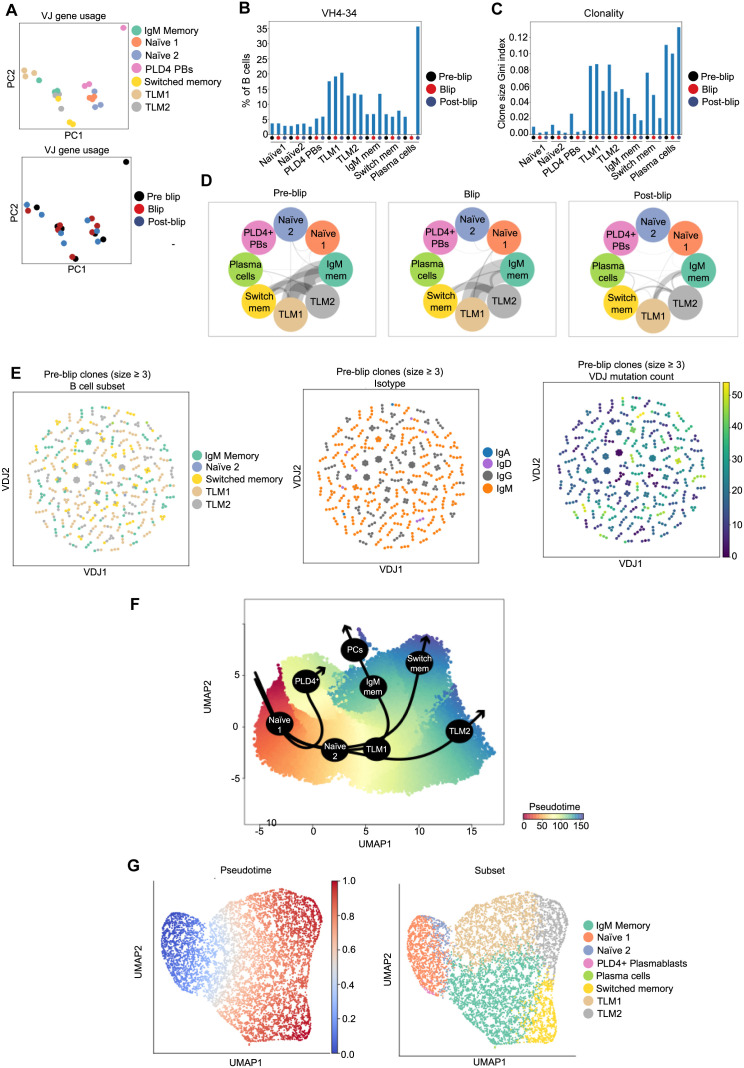
TLM subsets are heterogenous and display innate-like B cell characteristics. **A:** PCA based on VJ gene usage by elite controller B cells coloured by B cell subset and timepoint. **B:** Bar chart showing percentage of B cells using IGHV4-34 segment within each subset and timepoint. **C:** Bar chart showing clonality (clone size Gini index) of each B cell subset stratified by timepoint. **D:** Circos-style plots depicting clonal overlap between each B cell subset pair stratified within each timepoint. The thickness of the grey connecting line between circles reflects the number of overlapping BCR clonotypes. **E:** Single-cell BCR network plots of all pre-blip clones consisting of at least three cells, coloured by B cell subset annotation (left), heavy chain isotype class use (middle) and mutation count (right). Each circle/node represents a single B cell with a corresponding set of BCR(s). Cells belonging to the same clone are connected with a black line. **F:** UMAP visualization of all B cells described in Fig 4, coloured by GEX-based pseudotime and overlayed by inferred developmental lineages (black arrows), generated by slingshot package. **G:** UMAP generated by the BCR-seq-based pseudobulk VDJ trajectory analysis (dandelion package) of B cell dataset described in Fig 4, coloured by calculated pseudotime and B cell subset annotation.

To investigate the clonal relationship and potential differentiation pathway of annotated B cell subsets (in particular, TLMs) we utilised the single-cell BCR-seq data generated in the previous experiment. A principal component analysis (PCA) based on VJ gene usage revealed that TLM1 cells across all three timepoints clustered together and separate from other B cell subsets (Fig 5A). This was driven mainly by a high use of IGHV4–34 segment (Fig 5B), which was also observed, albeit to a lesser extent, in the TLM2 subset (Figs 5B and S5). IGHV4–34 is often found in self-reactive and lymphoma B cells [38–41]. Surprisingly, TLM1 subset also appeared one of the most clonal groups apart from plasma cells, the analysis of which was likely less reliable given their small cell count (Fig 5C). Overall, the high frequency of IGHV4–34 segment use and clonal appearance of the TLM1 subset despite a relatively low SHM burden (Fig 4K) indicates that this subset is likely enriched for innate-like B cells [42]. The clonal overlap analysis showed the strongest link between TLM1 and IgM MBCs, reaching its maximum during the blip (Fig 5D). Interestingly, TLM2 cells overlapped equally with TLM1, IgM memory and switched memory subsets, suggesting a likely heterogeneous origin of these cells (Fig 5D). Indeed, we observed many clones composed of cells from different B cell subsets, although individual clones seemed mostly homogenous in their isotype use (Fig 5E). TLM2 cells within some mixed (cross-subset) clones had the highest VDJ mutation count, suggesting them as one possible differentiation endpoint for TLM1(Figs 5E and S5). To explore the differentiation pathways of the B cell subsets further, we used the Slingshot package to infer pseudotime and developmental trajectories and identified four distinct developmental lineages (Fig 5F). PLD4 + plasmablasts differentiated directly from naïve B cells, which is consistent with their proposed extrafollicular origin. Interestingly, the TLM1 subset appeared to be the branching point for three distinct lineages (IgM memory, switched memory and TLM2). Although TLM1 formed a cluster of transcriptomically similar B cells, they are still heterogenous and poised for different differentiation pathways. These cells likely include innate-like B cells that tend to progress into TLM2, and naïve B cells differentiating into memory cells (either IgM or switched). Finally, we replicated these GEX-based pseudotime analysis findings also using BCR-seq-based pseudobulk VDJ trajectory analysis (Fig 5G). In conclusion, our investigation into the origins and differentiation potential of TLM cells through BCR and 5’GEX data analysis revealed high IGHV4–34 usage, and high clonality with lower SHM burden compared to MBCs. However, both TLM subsets also contained cells from IgM memory and switched memory clones, indicating the potential for TLM1 cells to differentiate into conventional MBCs whilst TLM2 appeared to be the final differentiation endpoint.

## Discussion

Our study provides insights into B cell function during HIV infection in an individual elite controller who experienced a brief episode of low-level viremia, with a particular focus on TLMs that are observed in HIV-associated B cell dysfunction. Through bulk BCR sequencing of circulating B cells from 5 PLWH on ART or with spontaneous control of viremia, we observed that TLMs exhibited significantly lower SHM burden and lower diversity compared to other MBC subsets. This suggested TLMs in PLWH are formed either in an early differentiation state or a blockage in antigen-driven evolution and potentially represent an innate-like phenotype. Hence, our initial data suggested that in the index participant some TLMs may serve as precursors for more mature B cell subsets while others appear to be in a terminal differentiation state. To test this hypothesis we used multi-omics approaches to investigate the total B cell and TLM response to HIV viremia this unique case of an elite controller who experienced a transient HIV viral blip. We found that their TLM population was heterogenous, with some cells exhibiting innate-like B cell features, others forming a precursor population for activated and resting MBCs and some appearing to have reached a differentiation endpoint. The extent to which these different aspects of TLMs are found in other PLWH, including those on ART or with uncontrolled viremia, and in HIV-negative controls remains to be determined. Although notably other studies outside of HIV have identified TLMs arising as part of a normal vaccination response [43] and also observed heterogeneity within this subset [44]. Similarly, it is important to note that the expansion of the TLM compartment in other disease states including other chronic infections and autoreactivity may well occur in a divergent way. For example, it has been observed that mAbs cloned from TLMs against malaria antigens had a higher SHM burden than those from other subsets [11], suggesting their development can be antigen driven. This may represent differences in the MBC responses to different pathogens or be a more generalised phenomenon that occurs when antigen burden expands exponentially in any untreated infection.

In our index participant we observed an increase in anti-Env IgG and associated expansion of Env^+^ IgG^+^ MBCs without significant changes in TLM abundance during the viral blip, where viral load reached only 600 copies/mL. Interestingly, the Env^+^ MBCs displayed a predominantly non-TLM phenotype, clustering transcriptionally with resting and activated MBCs. This contrasts with previous studies of chronic HIV viremia, where Env^+^ cells were enriched for TLMs and total TLMs were suggested to contribute to overall immune dysfunction [7,9,45]. While this difference could be unique to our index participant, the minimal expansion of TLMs during the viral blip described here suggests that such short-lived increases in viral load do not drive the generation of dysfunctional TLMs as seen in chronic infection but instead support the production of functional, antigen-specific MBCs. This agrees with recent studies describing atypical MBCs playing a useful part in an immune response [44]. Moreover, this finding is in line with the study by Cooper et al. that showed that during acute LCMV infection in mice there are more antigen-specific MBCs and fewer CD11c^+^Tbet^+^ DN B cells than in chronic LCMV infection [21]. Combined, these observations are encouraging for the use of antiviral antibodies against HIV, which necessitates a treatment interruption, although the precise viral load at which TLMs are expanded remains to be determined.

However, our index participant did have an identifiable population of TLMs detected by classical cell surface expression markers, and by using sc-RNA sequencing we identified two distinct TLM subpopulations: TLM1 and TLM2. The former were predominantly IgM-biased, less mutated and expressed inhibitory receptors at a lower level than TLM2. Pseudotime analysis suggested that, in this individual, TLM1 is likely a precursor population, with the potential to progress into more mature B cell states (either classical memory or TLM2). In contrast, TLM2 cells from this individual, which were predominantly IgG^+^, appeared to represent a terminal differentiation state. The TLM2 gene expression pattern closely resembled the expanded TLM population described previously during chronic HIV infection with high viremia [9,20] and the double-negative (DN2) population described in healthy controls [46]. In these previous studies, these subsets were proposed to be the differentiation endpoints in agreement with our findings. Although our study was just in one individual, the identification of multiple TLM subsets is consistent with previous observations using scRNA sequencing technologies [46].

One intriguing observation from the individual studied here was the relatively frequent use of IGHV4–34 in TLM1 and to a lesser degree in TLM2 population. This VH gene is often associated with self-reactive and innate-like B cell phenotypes [42,47]. These heterogenous unconventional B cells have previously been characterized by their innate sensing, T-independent response capacity and spontaneous production of germline-encoded natural antibodies that play a role in first-line defence and tissue homeostasis (e.g., apoptotic cell disposal) [48]. The prime examples of innate-like B cells are marginal-zone B cells and B-1 cells, the existence of which in humans remains controversial [49]. Innate-like B cells express inhibitory BCR co-receptors to allow their unique tonic BCR signalling and activation pattern. In line with this, the Hallmark GSEA analysis of the MBCs of our index participant revealed that, unlike other subsets, TLMs were in a state of persistent activation and BCR signalling across all three timepoints (highest in the pre-blip phase) suggesting an innate-like character. However, our study demonstrated that the TLM subset in this individual contained not only innate-like B cells but also cells from the conventional MBC lineages, highlighting the complexity of the whole TLM compartment and the need to understand the proportion and roles of these two types of TLM across greater numbers of PLWH in relation to their viral load status in future studies.

Another B cell subset with innate-like features identified by this study of our index participant who briefly lost their spontaneous control of their HIV viral load, were PLD4 + plasmablasts that emerged significantly with the viral blip, most likely via the extrafollicular route [50–52]. PLD4 is Phospholipase D4 an RNA/DNA exonuclease located in endo-lysosomes of plasmacytoid dendritic cells that can be upregulated in B cells upon TLR7/9 signalling [53]. Recently, self-reactive PLD4 + plasmablast enrichment was found in patients with SLE and were shown to phenotypically overlap with T-bet + DN2 B cells in SLE [53]. The PLD4 + plasmablast subset had the strongest type I IFN signature in the post-blip phase in this individual, however, any significance in the anti-HIV antibody response remains to be explored during chronic HIV infection and those with suppressed viremia across multiple individuals.

The primary limitation of our study is that our detailed analysis was conducted in a single elite controller experiencing a transient viral blip, which limits the generalizability of our findings. However, the temporal samples from this individual did allow us to investigate the effects of an acute and small HIV viral load increase and its impact on humoral immunity. Furthermore, while we observed key insights into B cell dynamics, the absence of sustained/increasing viremia in our index participant prevents us from fully exploring the mechanisms driving TLM expansion and dysfunction seen in chronic untreated HIV infection. Future studies involving larger cohorts and longitudinal data will be crucial to validate these findings and explore the pathways governing TLM development under different viral load conditions and when viral suppression is achieved by ART rather than spontaneous control. Additionally, our analysis of the viral sequences was constrained by the inability to obtain viral RNA (although we successfully obtained proviral DNA) during the blip, limiting the investigation of the viral dynamics driving the humoral response. Nevertheless, our use of genomic DNA was supported by multiple previous studies suggesting that provirus from genomic DNA does reflect recent viral integration sequences [54–56].

Overall, our study demonstrates that, in this unique individual living with HIV who experienced a viral blip, TLMs are a heterogeneous population significantly enriched for B cells with innate-like characteristics that are IFN not antigen driven. Two distinct TLM subsets (TLM1 and TLM2) were distinguished: TLM1 (T-bet^low^) cells retained the capacity for further differentiation, whereas TLM2 (T-bet^hi^) cells appeared to represent a terminal differentiation state. Our data show that such a small transient increase in HIV viral load, while capable of eliciting a strong serum response, did not in this individual drive the expansion of dysfunctional TLMs typically associated with chronic HIV infection. This is interesting in light of ongoing clinical trials using brief therapy interruptions where transient (albeit much higher) viral loads are observed.

Future studies should aim to understand the tipping point between viremia promoting a functional immune response (as in our index participant) versus inducing the emergence of dysfunctional B cell subsets and enhanced T cell destruction seen previously in chronic uncontrolled HIV-1. Understanding how the magnitude and duration of viremia are linked to B and T cell dysfunction will be informative for developing strategies for novel therapeutic interventions in HIV-1 infection, such as antibody treatment, that involve a period of ART cessation. For example, by determining at what level of transient viremia there exists a window for using protective/restorative therapies to modify the inflammatory environment and shelter MBCs from dysfunctional expansion. Moreover, larger studies that confirm the precise molecular signals and pathways that govern MBC differentiation and dysfunction in HIV could enable the development of vaccine adjuvants that preferentially induce a response dominated by resting MBCs rather than bystander activation increasing TLMs. Such vaccination strategies could be beneficial for use with routine vaccinations where PLWH have been reported to have weaker responses [2,4] as well as in other scenarios such as advanced age [57] or obesity [58] where inferior vaccine outcomes have been associated with expanded TLM populations.

## Materials and methods

### Ethics statement

The index participant was classified as exhibiting elite control based upon their largely stable low viral load (<100 copies/mL) over 13 years whilst not on ART (patient preference) prior to the viral blip. Samples from PLWH who had detectable viremia or non-ART mediated viral control were collected as part of a protocol approved by local research ethic committee (London – City & East REC 12/LO/1572). Samples from PLWH on ART (suppressed) and HIV-negative participant samples were previously collected and processed with ethical approval by South Central – Hampshire B (REC 19/SC/0423) [2]. Samples from chronic untreated HIV and HIV-negative volunteers were collected with the University of KwaZulu-Natal Biomedical Research Ethics Committee (BREC) approval (BREC/00001275/2020) and the Collection of Sputum, Urine and Blood samples (CUBS) cohort with BREC approval (BE022/13) respectively. Studies complied with all relevant ethical regulations for work with human participants and conformed to the Helsinki declaration principles and Good Clinical Practice (GCP) guidelines. All subjects enrolled into the studies provided written informed consent.

### PBMC isolation

Heparinised blood was isolated, diluted with an equal volume of PBS (Sigma Aldrich, Dorset, UK) and layered over 20 mL Histopaque-1077 (Sigma Aldrich). Peripheral blood mononuclear cells (PBMCs) and plasma were then isolated by centrifugation (400x g, 20 minutes, no brake). A 1x 15 mL aliquot and 2x 1 mL aliquots of plasma were recovered and stored at −80°C for future use. The mononuclear layer was then recovered, washed in 40 mL PBS and collected by centrifugation (400x g, 5 minutes). Cells were then washed in complete RPMI (RPMI-1640 media (Sigma Aldrich) supplemented with L-glutamine (Sigma Aldrich), penicillin/streptomycin (Sigma Aldrich) and 10% fetal bovine serum (FBS) (Sigma Aldrich)), pelleted by centrifugation (400x g, 5 minutes), resuspended in complete RPMI and counted using trypan blue exclusion. PBMC were cryopreserved in cryovials for future use by resuspension at 1x10^7^ cells/ml in FBS 10% DMSO (Sigma Aldrich) and stored at -80°C.

### Single Genome Amplification (SGA) of *env* genes

The Qiagen DNAEasy kit (Qiagen, Manchester, UK) was used to extract DNA from 1x107 PBMCs using the spin column protocol with some minor modifications. Cells were lysed for 30 minutes at 56°C rather than the recommended 10 minutes. A longer lysis step was found to lead to increased DNA yield. Additionally, a modification was made to the elution step, whereby 90μl of 5mM Tris-HCL (Sigma Aldrich) was dispensed onto the column, the column was then warmed to 40°C and left for 20 minutes prior to centrifugation at 13,000 RPM for 1 minute. HIV *env* single genomes were PCR amplified as previously described [17] using isolated genomic DNA as template and primers shown in Table 1. In brief, gDNA was diluted in PBS and nested PCR carried out using High Fidelity Platinum Taq DNA polymerase (Invitrogen, Paisley, UK) with primers Env5out and OMF19, followed by nested primers Env5in and Env3in [59,60]. PCR amplifications were carried out in 1 × High Fidelity Platinum PCR buffer, 2mM MgSO4, 0.2mM dNTPs, 0.2μM of each primer and 0.025 units/μL Platinum Taq, with a final reaction volume of 10 μL. The thermocycler protocol was as follows: 94°C for 2 minutes, followed by 35 cycles of 94°C for 15 seconds, 55°C for 30 seconds and 68°C for 4 minutes, finishing with a 10-minute elongation step at 68°C. 2kb *env* amplicons were visualised by agarose gel electrophoresis on precast 96 well 1% agarose E-gels (Invitrogen). Wells where a single *env* amplicon was obtained then underwent one final round of PCR amplification using the Env Cloning Fwd and Env Cloning Rev primers which generate an *env* amplicon compatible with Gibson cloning into the psvIII expression vector. Conditions for the final PCR were unchanged although reactions were scaled up to 25 μL.

**Table 1:** Smart Seq 2 and Env sequencing primer sequences.

Primer name	Primer Sequence 5’-3’
Env 5’ out	TAGAGCCCTGGAAGCATCCAGGAAG
OFM19	GCACTCAAGGCAAGCTTTATTGAGGCTTA
Env 5’ in	CACCTTAGGCATCTCCTATGGCAGGAAGAAG
Env 3’ in	GTCTCGAGATACTGCTCCCACCC
Env Cloning Fwd	ANTTGTGGGTYACAGTHTATTATGGGGTAC
Env Cloning Rev	TCGTCCCAGATAAGTGCCAAGGATCCAYCC
A_5allspl	AAGAAGCGGAGACAGCGACGAAGA
B_179	CACATGGCTTTAGGCTTTGATCCCAT
C_178	ATGGTAGAACAGATGCATGAGGATATAAT
D_180	TGAGTTGATACTACTGGCCTAATTCCATGTG
E_V3Fin	GAACAGGACCATGTACAAATGTCAGCACAGTACAAT
F_mf169	TGATGGGAGGGGCATACAT
G_18	TGCAGAATAAAACAATTTATAAACATGTGGC
H_182	TGTATTAAGCTTGTGTAATTGTTAATTTCTCT
J_mf159	CTGGAACAGATTTGGAATAACATGACCT
K_pr10	TTTTGACCACTTGCCACCCATCTTATAGC
Template Switching Oligonucleotide (TSO) (note r = ribo, +=Locked Nucleic Acid)	AAGCAGTGGTATCAACGCAGAGTACATrGrG + G
Oligo-dT30VN	AAGCAGTGGTATCAACGCAGAGTACTTTTTTTTTTTTTTTTTTTTTTTTTTTTTTVN

### Envelope cloning

*env* PCR amplicons were purified using the QIAquick PCR purification kit (Qiagen) following the manufacturer’s recommendations. PsvIII vector containing the HXB2 *env* sequence was digested with the BamHI-HF and KpnI-HF restriction enzymes (NEB, Hitchin, UK) at 37°C for 3 hours in 1x CutSmart buffer (NEB). The digested plasmid was then visualised by agarose gel electrophoresis and the digested plasmid purified using the QIAquick Gel Extraction Kit (Qiagen) following the manufacturer’s recommendations. Purified inserts were then cloned into the digested psvIII expression vector via Gibson assembly. Briefly, 100 ng of digested psvIII vector was incubated with a 3-fold molar excess of PCR insert in 1x NEBuilder HiFi DNA Assembly Master Mix (NEB) for 2 hours at 50°C. NEB 10-beta Competent *E. coli* (High Efficiency) were subsequently transomed with the assembly mix and incubated on LB-Agar ampicillin selection plates overnight at 37°C. The following day single colonies were upscaled to 1 mL LB-Broth ampicillin culture for 6 hours at 30°C, 200 RPM. 2 μL of culture was then added to 48 μL H_2_O and placed at -80°C for 1 hour before incubation at 95°C for 15 minutes. The resulting supernatant was then used as template for a PCR reaction with the Env Cloning Fwd and Env Cloning Rev primers as described above. *env* amplicons were visualised by agarose gel electrophoresis on precast 96 well 1% agarose E-gels (Invitrogen). Wells which contained *env* amplicons were upscaled to 100 mL LB-Broth ampicillin culture 30°C, 200 RPM for 16 hours and then plasmid DNA isolated using the plasmid plus midi sample kit (Qiagen).

### Envelope Sequencing and Phylogenetic analysis

Cloned *env* amplicons underwent Sanger-sequencing using the A-K sequencing primers. Sequence reads were trimmed at the 5′ and 3′ ends until base calls consistently reached a quality score ≥30, resulting in 99.9% accuracy of base calls. Contigs were then generated using SnapGene Software (www.snapgene.com) by alignment to the HXB2 *env* sequence. *env* contigs were trimmed resulting in amplicons from amino acid 35 to amino acid 683 (HXB2 numbering). *env* sequences were then aligned at the protein sequence level using MUSCLE v.3.8.31 [61] and mapped back to nucleotide, with minor manual adjustment. 2 clade B sequences (NL4–3 and B41) were included for reference and 2 clade A sequences (BG505 and 92UG029.EC1) from the Los Alamos HIV Sequence Database (http://www.hiv.lanl.gov/) were included to serve as an outgroup. A maximum likelihood phylogeny was estimated using IQ-tree software [62], with the general time-reversible nucleotide substitution model and gamma-distributed rate heterogeneity. Clade support was estimated from 1,000 nonparametric bootstrap replicate datasets. The maximum likelihood phylogeny was rooted on the outgroup branch and visualized using FigTree v.1.4.4 (http://tree.bio.ed.ac.uk/software/figtree/).

### Soluble Envelope protein production

Three diverse *env* sequences from the maximum likelihood phylogeny: 1A3, 1D5 and 2E4 were selected to be expressed as soluble stabilized proteins. To allow for stabilized expression, SOSIP mutations were introduced into the sequences as described by Sanders et al [28] and sequences ordered as synthetic Gene Fragments with Gibson cloning compatible overhangs (Genewiz, Bishop’s Stortford, UK). p818 plasmid was digested with the BstBI and PstI restriction enzymes and gel extracted, *env* Gene Fragments were then cloned into the digested vector as described in the “envelope cloning” methods section. Stabilized Env proteins were expressed in HEK293F cells and purified with either PGT145 or 2G12 affinity chromatograph followed by size exclusion chromatography (SEC) using a HiLoad 16/600 Superdex pg200 (GE Healthcare) as described previously [28,63,64]. The Avi-tagged proteins were biotinylated using the BirA enzyme (Avidity, Colorado, USA) according to the manufacturer’s instructions.

### Pseudovirus production

HEK-293T cells were co-transfected with Env-expressing psvIII plasmid and a HIV Env deficient backbone plasmid (pSG3ΔEnv) at a mass ratio of 1:2 using polyethyleneimine (PEI) max (Polysciences, Bergstrasse, Germany) at a DNA: PEI mass ration of 1:3 and incubated for 48 hours at 37°C 5% CO_2_. The pseudovirus containing supernatant was then harvested and passed through a 0.45 μm syringe filter (Sigma Aldrich), aliquoted and stored at -80°C. Subsequently, frozen pseudovirus was titrated using the HeLa-TZM-bl reporter cell assay with the Bright-Glo Luciferase assay kit (Promega, Hampshire, UK) and the 50% tissue culture infectious dose (TCID_50/_mL) calculated.

### *In vitro* neutralization assays

Pseudovirus neutralization assays were carried out using the 96-well plate TZM-bl cell-based assay described by Sarzotti-Kelsoe et al [30], with Bright-Glo luciferase reagent (Promega) and final readout analysed using a BioTek Synergy H1 plate reader (Agilent technologies, Manchester, UK). Briefly, plasma samples were heat-inactivated by incubation at 56°C for 1 h and then titrations of plasma starting at 1:100 incubated with luciferase encoding pseudovirus (at 200 TCID_50_/mL) in duplicate for 1 hour at 37°C 5% CO_2_. 10,000 HeLa TZM-bl cells were then added to each well and incubated for 48 hours at 37°C 5% CO_2_ before supernatants were removed and luciferase readout used as an indicator of infectivity. 100% infectivity was defined by wells which contained virus and TZM-bl cells only, with no antibody present.

### Semi-quantitative total Ig ELISA

Half-area 96-well MaxiSorp plates (VWR, Leicestershire, UK) were coated with either 25 μL goat anti-human F(ab)’2 (1:1,000) (Jackson immunoResearch, Cambridgeshire, UK) in PBS for total IgG plates, or 25 μL mouse anti-human Ig κ light chain and mouse anti-human Ig λ light chain (1:200 each) (Becton Dickinson Biosciences, Wokingham, UK) in PBS for total IgG3 plates, and incubated overnight at 4°C. Plates were washed with PBS-T and blocked with 100 μL assay buffer (PBS, 3% BSA, 0.05% Tween-20) for 1 hour at room temperature. 25 μL of serially diluted plasma (1:100–1:10^7^) in duplicate or known concentrations of IgG or IgG3 standard in triplicate (Sigma Aldrich) were then applied to the plates. Plates were washed with PBS-T and 25 μL of detection antibody diluted in assay buffer added to each well: goat anti-human IgG-AP (1:1,000) (Sigma Aldrich) or goat anti-human IgG3-AP (1:1,000) (Cambridge Bioscience, Cambridge, UK) and incubated for 1 hour at room temperature. Subsequently, plates were washed 6 times with PBS-T and 25 µL of colorimetric alkaline phosphatase substrate added (Sigma Aldrich). Absorbance was measured at 405 nm after 60 minutes. Total IgG or IgG3 concentrations in plasma were then calculated based on interpolation from the IgG or IgG3 standard curves using a four-parameter logistic (4PL) regression curve fitting model.

### Semi-quantitative Env ELISA

Columns 1–9 of half-area 96-well MaxiSorp plates were coated overnight at 4°C with streptavidin in PBS (2 µg/ml per well in 25 µL) (Sigma Aldrich). Columns 10–12 were coated with either 25 μL goat anti-human F(ab)’2 (1:1,000) in PBS for IgG standard curves, or 25 μL mouse anti-human Ig κ light chain and mouse anti-human Ig λ light chain (1:200 each) in PBS for IgG3 standard curves. Plates were then washed with PBS-T and blocked with 100 μL assay buffer (PBS, 3% BSA, 0.05% Tween 20) for 1 hour at room temperature. 25 μL of biotinylated Env protein (2 µg/ml) in assay buffer was then added to streptavidin coated wells and 25 µL of assay buffer added to wells in columns 10–12 for 2 hours at room temperature. Following washing 25 μL of serially diluted plasma (1:100 to 1:10^7^) in duplicate or known concentrations of IgG or IgG3 standard in triplicate were then applied to the plates. Plates were washed with PBS-T and 25 μL of detection antibody diluted in assay buffer added to each well: goat anti-human IgG-AP (1:1,000) or goat anti-human IgG3-AP (1:1,000) and incubated for 1 hour at room temperature. Subsequently, plates were washed 6 times with PBS-T and 25 µL of colorimetric alkaline phosphatase substrate added. Absorbance was measured at 405 nm after 60 minutes. Env reactive IgG or IgG3 concentrations in plasma were then calculated based on interpolation from the IgG or IgG3 standard curves using a four-parameter logistic (4PL) regression curve fitting model.

### Flow cytometry and single cell sorting

Fluorescence-activated cell sorting (FACS) of Env reactive cryopreserved PBMCs was performed on a BD FACS Melody (BD Biosciences). Alternatively, memory B cell subsets were sorted using a BD FACS Aria II (BD Biosciences). To generate fluorescently tagged Env probes 2 μg of biotinylated Env protein was incubated with either 0.5 μg streptavidin-conjugated PE (BD Biosciences) or 0.5 μg streptavidin-conjugated BV786 (BD Biosciences) for 30 minutes. Cryopreserved PBMC were thawed on ice and incubated with 100 μL of Zombie Live/Dead dye (diluted 1:400 in PBS) (Biolegend, London, UK) for every 5x10^6^ cells at room temperature for 20 minutes. The dye was then quenched by addition of complete RPMI, cells collected by centrifugation (400x g, 10 minutes) and then washed once in PBS. Cells were then incubated with specific combinations of the phenotyping antibodies + /- biotinylated tetramers listed in Table 2 in a final volume of 100 μL in PBS (concentrations used were for every 5x10^6^ cells). Compensation controls were prepared according to manufacturer’s instructions using Anti-Mouse Ig, κ and negative control compensation particles (BD Biosciences). For Smart-Seq 2 experiments single B cells were gated according to the following successive gates: lymphocyte, single cells, CD19^+^ Aqua live/Dead^-^ CD4^-^ and IgM^-^ IgG^+^. From this population 1x 96 well plate of each of the following subsets was sorted: CD21^-^ CD27^-^ (TLM cells), CD21^-^ CD27^+^ (activated memory cells) and CD21^+^ CD27^+^ (resting memory cells). Alternatively, antigen specific cells were sorted based on positivity for either the PE-tagged or BV786-tagged Env probes. All cells were sorted using “Yield” purity mode into chilled 96-well plates (VWR) containing 4.4 μL of lysis buffer composed of oligo-dT (5′-AAGCAGT GGTATCAACGCAGAGTACT30VN-3′) (1 μM), 0.4% Triton X-100 (Sigma Aldrich), dNTPs (1mM) (Thermo Fisher, Paisley, UK), and RNAse inhibitor (4 units) (Takara Bio, London, UK). After each plate was filled, it underwent centrifugation at 400x g for 1 minute and placed on dry ice immediately before storage at −80°C. For bulk BCR sequencing experiments single B cells were gated according to the following successive gates: lymphocyte, single cells, CD19^+^ Aqua live/Dead^-^ CD14^-^ CD3^-^, CD20^+^ CD38^lo^, IgD^-^ and either IgM^-^ IgG^+^ or IgM^+^ IgG^-^. From this population the following subsets were sorted: CD21^-^ CD27^-^ (TLM cells), CD21^-^ CD27^+^ (activated memory cells) and CD21^+^ CD27^+^ (resting memory cells). Bulk cell populations were sorted using “Yield” purity mode into chilled 1.5 mL tubes containing lysis buffer (0.3% NP40 and ~6.67mM RNaseOUT). After sorting of each donor was complete each 1.5 mL tube underwent centrifugation at 400x g for 1 minute and was placed on dry ice immediately before storage at −80°C. Data was analyzed on FlowJo v10 (BD).

**Table 2:** B cell phenotyping flow cytometry and CITE-Seq panels. Where suppliers do not provide antibody concentrations the volume of antibody used (μL) is provided.

Marker	Fluorophore/Cite-Seq Barcode	Supplier	Clone	Isotype	Concentration (μg/mL)
CD3	BV510	Biolegend: 317332	OKT3	Mouse IgG2a, κ	1
CD14	BV510	Biolegend: 301842	M5E2	Mouse IgG2a, κ	1
CD4	BV510	BD: 562970	SK3	Mouse IgG1, κ	2 μL
CD19	FITC	Biolegend: 363008	SJ25C1	Mouse IgG1, κ	4
CD20	AF700	BD: 560631	2H7	Mouse IgG2a, κ	1 μL
CD21	PE-Cy7	BD: 561374	B-ly4	Mouse IgG1, κ	5
CD21	BV711	BD: 563163	B-ly4	Mouse IgG1, κ	1 μL
CD27	BV421	BD: 562514	M-T271	Mouse IgG1, κ	2.5
CD38	PE-Dazzle	BD: 562288	HIT2	Mouse IgG1, κ	0.5 μL
IgD	PE-Cy7	BD: 561314	IA6–2	Mouse IgG2a, κ	1 μL
IgM	APC-Cy7	Biolegend: 314519	MHM-88	Mouse IgG1, κ	1
IgG	APC	BD: 550931	G18-145	Mouse IgG1, κ	10 μL
IgG3	BV605	BD: 753735	HP6050	Mouse IgG1, κ	20
Env Probe 1	Strep - PE	BD: 554061	N/A	N/A	N/A
Env Probe 2	Strep - BV786	BD: 563858	N/A	N/A	N/A
CD4	GAGGTTAGTGATGGA	Biolegend: 344651	SK3	Mouse IgG1, κ	0.25
CD8	GCGCAACTTGATGAT	Biolegend: 344753	SK1	Mouse IgG1, κ	0.5
CD19	CTGGGCAATTACTCG	Biolegend: 302265	HIB19	Mouse IgG1, κ	0.5
CD21	AACCTAGTAGTTCGG	Biolegend: 354923	Bu32	Mouse IgG1, κ	0.5
CD27	GCACTCCTGCATGTA	Biolegend: 302853	O323	Mouse IgG1, κ	0.5
IgD	CAGTCTCCGTAGAGT	Biolegend: 348245	IA6–2	Mouse IgG2a, κ	0.5
IgM	TAGCGAGCCCGTATA	Biolegend: 314547	MHM-88	Mouse IgG1, κ	1
IgG	CTGGAGCGATTAGAA	Biolegend: 410727	M1310G05	Rat IgG2a, κ	0.25
FcRL5	TCACGCAGTCCTCAA	Biolegend: 340309	509f6	Mouse IgG2a, κ	0.5
CD85j	CCTTGTGAGGCTATG	Biolegend: 333725	GHI/75	Mouse IgG2b, κ	1
Isotype Control	GCCGGACGACATTAA	Biolegend: 400187	MOPC-21	Mouse IgG1, κ	1
Isotype Control	CTCCTACCTAAACTG	Biolegend: 400293	MOPC-173	Mouse IgG2a, κ	0.5
Isotype Control	ATATGTATCACGCGA	Biolegend: 400381	MPC-11	Mouse IgG2b, κ	1
Isotype Control	AAGTCAGGTTCGTTT	Biolegend: 400577	RTK2758	Rat IgG2a, κ	0.25

### cDNA generation, library preparation, and sequencing (Smart-seq2)

The Smart-seq2 protocol [32] was utilised with some minor adjustments for working with lymphocytes. In brief, 96-well plates containing single cell lysates were thawed on ice. Each well then received 5.4 μL of reverse transcription mix containing Superscript II reverse transcriptase (50 units) (Thermo Fisher), RNAse inhibitor (10 units) (Tarka Bio), 5X first strand buffer (Thermo Fisher), DTT (5mM) (Thermo Fisher), betaine (1M) (Sigma), MgCl2 (12mM) (Sigma Aldrich), Nuclease-free water (Thermo Fisher) and template switching-oligo (5′- AAGCAGTGGTATCAACGCAGAGTACATrGrG + G-3′) (1μM). Reverse transcription (RT) was carried out for 90 minutes at 42°C followed by 10 cycles of: 50°C for 2 minutes and 42°C for 2 minutes to allow for completion/continuation of RT and template-switching, then finally 70°C for 15 minutes to terminate the reaction. Each well then received 15 μL of PCR mix containing Nuclease-free water, and 2x HiFi HotStart ReadyMix (Roche, Welwyn Garden City, UK). Cells were then subjected to the following thermocycler protocol: 98°C for 3 minutes, 24 cycles of: 98°C for 20 seconds, 67°C for 20 seconds, and 72°C for 6 minutes, then finally 72°C for 5 minutes. Following amplification, 0.8 × AMPure XP beads (Beckman Coulter, High Wycombe, UK) were used to purify cDNA and remove primer dimers. The size distribution of cDNA in each well as well as the concentration of cDNA in the 500–5000 bp range was subsequently determined using a High Sensitivity D5000 ScreenTape kit (Agilent Technologies) and readout analysed on an Agilent 4200 TapeStation System. Cells that exhibited cDNA profiles indicative of degraded RNA were excluded from subsequent library preparation. The cDNA from remaining wells were diluted to approximately 0.5 ng/μL before carrying out the Nextera XT DNA library preparation (Illumina, Cambridge, UK) following manufacturer’s instructions. 0.6 × AMPure XP beads (Beckman Coulter) were used to purify cDNA and remove primer dimers. Libraries were then quality checked and quantified once again using the High Sensitivity D5000 ScreenTape system and then libraries were pooled at equimolar concentrations for sequencing. In total, 443 cells were sequenced across two runs using 2 × 75 bp NextSeq 500 High Output v2.5 kits on an Illumina NextSeq 500.

### CITE-seq staining for single-cell proteogenomics

Frozen PBMC samples were thawed at 37°C in a water bath. Ice cold compete RPMI was added slowly to the cells before centrifuging at 300x *g* for 5 min. This was followed by a wash in 5 ml complete RPMI. The PBMC pellet was collected, resuspend in ice cold PBS and the cell number and viability were determined using Trypan Blue. A portion of the PBMC from each timepoint was then placed in 1.5 mL tubes and placed on ice, whilst the remaining PBMC underwent magnetic cell sorting (Miltenyi Biotec, Bergisch Gladbach, Germany) to purify the B cell populations. After isolation B cells were mixed with non-purified PBMC at a 3:1 ratio. The cells were then resuspend in 100 μL of mastermix containing CITE-Seq antibodies (Table 3) for 30 min at 4°C. cells were then washed three times by centrifugation at 500x *g* for 5 min at 4°C. PBMCs were counted again and processed immediately for 10x 5′ single cell capture (Chromium Next GEM Single Cell V(D)J Reagent Kit v2 with Feature Barcoding technology for cell Surface Protein-Rev F protocol) (10x genomics, Pleasanton, CA, USA). Three lanes of 20,000 cells from each timepoint were loaded onto a 10x chip.

**Table 3:** Bulk BCR sequencing primers.

Primer Name	Stage	Primer sequence
PT020_R2	RT	GGAGTTCAGACGTGTGCTCTTCCGATCTNNNNNNNNNNNNNGCCAGGGGGAAGACCGATGGG
PT021_R2	RT	GGAGTTCAGACGTGTGCTCTTCCGATCTNNNNNNNNNNNNNCCGACGGGGAATTCTCACAGGAGACGAGGGGGAAAAG
PT056_R2	PCR1/PCR2	GGAGTTCAGACGTGTGCTCT
5’ L-VH 1 (H1F)	PCR1	ACAGGTGCCCACTCCCAGGTGCAG
5’ L-VH 3 (H1F)	PCR1	AAGGTGTCCAGTGTGARGTGCAG
5’ L-VH 4/6 (H1F)	PCR1	CCCAGATGGGTCCTGTCCCAGGTGCAG
5’ L-VH 5 (H1F)	PCR1	CAAGGAGTCTGTTCCGAGGTGCAG
VH2A_R1 (H1F/H2F)	PCR2	CTACACTCTTTCCCTACACGACGCTCTTCCGATCTATGGACAYACTTTGYTCCAC
VH2B_R1 (H1F/H2F)	PCR2	CTACACTCTTTCCCTACACGACGCTCTTCCGATCTATGGACACACTTTGCTACAC
5’Agel VH1/5_R1	PCR2	CTACACTCTTTCCCTACACGACGCTCTTCCGATCTGAGGTGCAGCTGGTGCAG
5’Agel VH3_R1	PCR2	CTACACTCTTTCCCTACACGACGCTCTTCCGATCTGAGGTGCAGCTGGTGGAG
5’Agel VH4_R1	PCR2	CTACACTCTTTCCCTACACGACGCTCTTCCGATCTCAGGTGCAGCTGCAGGAG
5’Agel VH3–23_R1	PCR2	CTACACTCTTTCCCTACACGACGCTCTTCCGATCTGAGGTGCAGCTGTTGGAG
5’Agel VH4–34_R1	PCR2	CTACACTCTTTCCCTACACGACGCTCTTCCGATCTCAGGTGCAGCTACAGCAG
PT701.2	PCR3	CAAGCAGAAGACGGCATACGAGATTCGCCTTAGTGACTGGAGTTCAGACGTGTGCTCT
PT702.2	PCR3	CAAGCAGAAGACGGCATACGAGATCTAGTACGGTGACTGGAGTTCAGACGTGTGCTCT
PT703.2	PCR3	CAAGCAGAAGACGGCATACGAGATTTCTGCCTGTGACTGGAGTTCAGACGTGTGCTCT
PT704.2	PCR3	CAAGCAGAAGACGGCATACGAGATGCTCAGGAGTGACTGGAGTTCAGACGTGTGCTCT
PT705.2	PCR3	CAAGCAGAAGACGGCATACGAGATAGGAGTCCGTGACTGGAGTTCAGACGTGTGCTCT
PT706.2	PCR3	CAAGCAGAAGACGGCATACGAGATCATGCCTAGTGACTGGAGTTCAGACGTGTGCTCT
PT707.2	PCR3	CAAGCAGAAGACGGCATACGAGATGTAGAGAGGTGACTGGAGTTCAGACGTGTGCTCT
PT708.2	PCR3	CAAGCAGAAGACGGCATACGAGATCCTCTCTGGTGACTGGAGTTCAGACGTGTGCTCT
PT709.2	PCR3	CAAGCAGAAGACGGCATACGAGATAGCGTAGCGTGACTGGAGTTCAGACGTGTGCTCT
PT710.2	PCR3	CAAGCAGAAGACGGCATACGAGATCAGCCTCGGTGACTGGAGTTCAGACGTGTGCTCT
PT711.2	PCR3	CAAGCAGAAGACGGCATACGAGATTGCCTCTTGTGACTGGAGTTCAGACGTGTGCTCT
PT712.2	PCR3	CAAGCAGAAGACGGCATACGAGATTCCTCTACGTGACTGGAGTTCAGACGTGTGCTCT
PT501.3	PCR3	AATGATACGGCGACCACCGAGATCTACACTAGATCGCTCTTTCCCTACACGACG
PT502.3	PCR3	AATGATACGGCGACCACCGAGATCTACACCTCTCTATTCTTTCCCTACACGACG
PT503.3	PCR3	AATGATACGGCGACCACCGAGATCTACACTATCCTCTTCTTTCCCTACACGACG
PT504.3	PCR3	AATGATACGGCGACCACCGAGATCTACACAGAGTAGATCTTTCCCTACACGACG
PT505.3	PCR3	AATGATACGGCGACCACCGAGATCTACACGTAAGGAGTCTTTCCCTACACGACG
PT506.3	PCR3	AATGATACGGCGACCACCGAGATCTACACACTGCATATCTTTCCCTACACGACG
PT507.3	PCR3	AATGATACGGCGACCACCGAGATCTACACAAGGAGTATCTTTCCCTACACGACG
PT508.3	PCR3	AATGATACGGCGACCACCGAGATCTACACCTAAGCCTTCTTTCCCTACACGACG

### Library generation and sequencing

The Chromium Next GEM Single Cell 5′ V(D)J Reagent Kit (V2 chemistry) with Feature Barcoding technology for cell surface proteins was used for single-cell RNA-seq library construction for all samples. GEX and V(D)J libraries were prepared according to the manufacturer’s protocol (10x Genomics) using individual Chromium i7 Sample Indices. The cell surface protein libraries were created according to the manufacturer’s protocol. 5’GEX, V(D)J and cell surface protein indexed libraries were pooled and sequenced on a NovaSeq 6000 S4 Flow cell (paired-end, 150 bp reads) aiming for a minimum of 50,000 paired-end reads per cell for 5’GEX libraries and 10,000 paired-end reads per cell for V(D)J and cell surface protein libraries.

### Bulk sorted BCR library construction

Bulk sorted MBC subsets (in lysis buffer) were collected from -80°C and incubated at room temperature to thaw the samples. The lysate was collected at the bottom of the tube by centrifugation. To purify the RNA, 1.7X volumes of room temperature RNAClean XP RNA purification beads were used (Beckman Coulter), binding RNA to beads for 20 minutes at room temperature. Mixtures were magnetically separated and then washed with fresh 80% ethanol. They were then air-dried before and eluted using molecular biology grade water (Ambion) for 10 minutes at room temperature. Clarified RNA containing supernatant was transferred to a clean tube.

To generate bulk BCR libraries from each MBC type, 15µL per RNA sample was combined with dNTPs (0.5mM, Thermo Fisher) and C-region specific primer (100nM) (Table 3) and molecular biology grade water, to a final volume of 17.75µL. Reverse transcription was performed using the SuperScript IV First-Strand Synthesis System, following manufacturer’s recommendations in a final volume of 25µL, using C-region specific primers (100nM) that contained UMIs and the Illumina read 2 site (Table 3). An IgG-specific C-region primer was used for the bulk sorted IgG^+^ MBC populations and an IgM-specific primer was used for the bulk sorted IgM^+^ MBC populations. This enabled us to perform heavy chain only bulk BCR sequencing. The reaction proceeded at 50°C for 45 minutes, prior to enzyme inactivation at 80°C for 10 minutes, followed by degradation of RNA: cDNA hybrids using RNaseH (New England Biolabs). cDNA product was purified using 1.0X AMPURE XP DNA purification beads (Beckman Coulter).

Eluted cDNA was supplemented with a mixture of leader sequence specific forward primers (H1F mix; 500nM), and a tag-specific reverse primer (PT056; 500nM), and 2X Q5 DNA polymerase Mastermix (New England Biolabs), to a final volume of 50µL, before undergoing PCR. This, and all subsequent PCRs, began with an initial denaturation at 98°C (50 seconds), and temperature then cycled between 98°C (10 seconds), 68°C (20 seconds) and 72°C (20 seconds) for a total 10 cycles, before a final extension at 72°C (120 seconds). The product was purified using 0.6X AMPURE XP DNA purification beads, and the eluted PCR product was supplemented with nested FWR1-specific primers containing the read 1 site (H2F mix; 500nM), a tag-specific reverse primer (PT056; 500nM) and Q5 DNA polymerase to a final volume of 50µL, which then underwent PCR. The product was purified using (0.6X) AMPURE XP DNA purification beads, and a final PCR was performed to add Illumina indexes (500nM each), again using Q5 DNA polymerase to the amplified DNA. PCR product was gel extracted using the QIAquick gel extraction kit (QIAGEN) to ensure homogenous size selection, prior to size and concentration quantification using the Agilent TapeStation 4150 (Agilent Technologies) and the Qubit 2.0 Fluorimeter (Thermo Fisher). Libraries were submitted for sequencing using the MiSeq platform, via UCL Genomics, using the following read lengths 325 bp, 8 bp, 8 bp and 275 bp for read 1, the two index reads, and read 2, respectively.

### Bulk BCR raw sequence processing

Raw sequencing reads (fastq) were processed using the Immcantation framework via singularity (version 4.4.0) [65,66]. Briefly, reads were filtered (phred score > 20), prior to primer and UMI region identification used to define coarse read groups. These were then more finely clustered using cd-hit-est [67] with a threshold of 90%. Similarly to other published works, we assume that the resulting groups represent reads from unique RNA molecules [68]). A consensus sequence was then generated per read group, and duplicate reads were collapsed to provide a single representative sequence. UMIs comprised of a single read were removed.

V, D and J regions were assigned using IgBLAST [69]. Annotated sequences were then partitioned into coarse clusters based on identical V, J and CDRH3 lengths and then finely clustered based on 90% CDRH3 amino acid identity. Amino acid mutations were identified with shazam [66].

For phylogenetic tree analysis, lineages containing multiple MBC subsets with at least 3 unique sequences were identified and processed using IgPhyML [70,71]. CDRH3 sequence was ignored for this analysis as per the developer’s recommendations.

### Bulk BCR bioinformatic analysis

Bulk BCR sequence analysis was carried out using R. Data manipulation and summarization was primarily performed using the tidyverse suite of packages [72], as well as foreach and doParallel for streamlining execution [73,74], and ape for basic analysis of trees [75]. The vegan package was used to calculate Shannon and Simpson diversity indices [76].

### De novo mutation calculation

Each lineage that contained multiple MBC subsets was first identified within each donor. Within these lineages, mutations were identified in BCR sequences by comparing each sequence to the lineage germline sequence. Next, BCR sequences underwent pairwise analysis to identify both shared and unique mutations for each sequence, and the proportion of unique mutations identified (unique mutations/ total mutations). The comparisons were then grouped based on the subsets compared (e.g., TLM—Resting, TLM—TLM as multiple sequences from the same subset were also compared), and the median average taken, to provide a single value per comparison, per lineage. Once computed for all lineages, this was then summarised by taking the mean average per comparison, to give a single value per donor, which underwent statistical comparison.

### BCR phylogenetic network analysis

To ascertain the evolutionary ‘potential’ of the MBC subsets, phylogenetic networks were created, using igraph [77]. To gain evolutionary information, lineages containing multiple MBC subsets were first identified and phylogenetic trees estimated using IgPhyML. From these trees, distance between tips (sum of branch lengths) was calculated using the distTips function from the adephylo R package [78], and assembled into a square matrix per lineage. To draw edges between related nodes, it was first necessary to define a distance threshold.

For this, the smallest tip-to-tip distance was identified for each tree, and a density curve created. For this curve, the lagged differences in y-axis value were divided by the lagged differences in x-axis, aiming to identify the position where the curve was most rapidly changing. This position was identified by taking the apex of the first identified peak (identified using the pracma package [79]), and this x-axis position was the selected cut-off. By choosing the position where the aggregated tip-to-tip distances were most rapidly changing, we aimed to identify a relevant distance where BCRs were linked to neighbouring BCRs and not linked to distant BCRs across multiple phylogenetic trees. This threshold was used to derive an adjacency matrix, whereby nodes that had a distance smaller than the calculated threshold were connected. The network was then directed by only allowing connections from nodes that had a smaller distance from the inferred germline node to those with a larger distance from the inferred germline node. Statistics were then calculated for each node in each network, namely the hub score (networkR; [80]) and the degree-ratio (igraph). Higher hub scores are achieved when a node has many outgoing connections and is further influenced by the quality of these downstream connections. Therefore, in our phylogenetic networks, nodes with higher hub scores reflect nodes with more close evolutionary relationships and indicates antigen driven SHM. The degree-ratio (number of outgoing connections divided by the number of incoming connections) was calculated using igraph and reflects the local environment of each node in the network. Statistical analysis was performed using Mann-Whitney U tests, via the ggpubr package (p > 0.05 not marked, p < 0.05 *, p < 0.01, **, p < 0.001 ***, p < 0.0001 ****) [81].

### Smart-Seq2 single-cell data analysis

Data were then processed using scanpy (v.1.9.8) [82] workflow with standard quality control steps; cells were filtered if number of genes >6000 or <600. Mitochondrial content was determined using scanpy.pp.calculate_qc_metrics function; cells with mitochondrial genes percentage <50% were retained for further analyses. Genes were retained if they were expressed by at least 2 cells. Gene counts for each cell were normalised to contain a total count equal to 10^6 counts per cell. This led to a working dataset of 301 cells. The top 2000 highly variable genes were selected based on Seurat v.3 algorithm (flavor = seurat_v3) with batch key “Sequencing_batch”. Highly variable genes were further refined by removing potentially confounding genes using the following search formula: ‘^HLA|^IG[HKL][VDJC]|^MT|^A[A-Z][0-9]|^B[A-Z][0-0]’. The number of principal components used for neighbourhood graph construction and dimensional reduction was set at 20. Data integration from both donors was performed using the bbknn algorithm [83]. Uniform Manifold Approximation and Projection (UMAP; v3.10.0) [84] was used for dimensional reduction and visualization with all parameters as per default settings in scanpy. For the assessment of transcriptional similarity between Env-reactive B cells and MBC reference subsets, Celltypist [85] probability scores were generated as per default settings.

### 10X data analysis

#### Data alignment and quantification.

Droplet libraries were processed using Cell Ranger v7.0.1; reads were aligned to the GRCh38 human genome (version refdata-gex-GRCh38-2020-A, 10X Genomics). CITE-seq UMIs were counted for GEX and ADT libraries simultaneously to generate feature-X droplet UMI count matrices.

#### CITE-seq data processing.

Filtered and raw Cell Ranger output count matrices were integrated into a MuData object and further processed using muon package pipeline v0.1.5 [86]. ADT counts for each protein were subjected to DBS (denoised and scaled by background) normalization with default settings. T/B cell doublets were removed using gene2filter if mutual expression at least two of the following markers was detected: CD19, CD4, CD8 (with positive expression thresholds set at 6, 12 and 12 for each respective marker).

#### Single-cell RNA seq quality control, normalization, embedding and clustering.

Scrublet (v0.2.3) [87] was applied to each sample to generate a doublet score. This generated binomial distribution with an automated (default) threshold for doublet definition. Combined raw data from all samples were processed using scanpy (v1.9.8) workflow with standard quality control steps: cells were filtered if number of genes >5000 or <400. Cells with mitochondrial and ribosomal genes percentage <10% and >5%, respectively, were retained for further analyses (total 48,602 cells post QC) Genes were retained if they were expressed by at least 3 cells. Data were normalized to contain a total count equal to 10^4^ per cell and log + 1 corrected. Top 2000 highly variable genes (HVG) were selected based on Seurat v.3 algorithm (flavor = seurat_v3) with batch key “sample” and refined by removing the following genes ‘^HLA|^IG[HKL][VDJC]|^MT|^A[A-Z][0-9]|^B[A-Z][0-0]|XIST’. The number of principal components used for neighbourhood graph construction and dimensional reduction was set at 20, number of neighbours at 10. Uniform Manifold Approximation and Projection (UMAP; v3.10.0) [84^]^ was used for dimensional reduction and visualization with all parameters as per default settings in scanpy. Clustering was performed using the Leiden algorithm with an initial resolution of 0.4. CellTypist v1.6.2 (model: Immune_All_Low.pkl) with majority voting was used for assisted initial cell annotation. Top 10 differentially expressed genes were calculated using the Wilcoxon rank-sum test with minimum log fold change (LFC) set at 2. For detailed B cell analysis, non-B/plasma cells were filtered out and the remaining dataset re-processed from HVG selection step again with the same setting but with Leiden clustering resolution 0.6.

#### Differential abundance testing.

The differential abundance of cell types across timepoints was determined using milopy (Milo framework) package (v0.1.0) standard workflow with default settings [35].

#### Cell-cell interactions inference.

Inferred number and strength of cell-cell interactions between all annotated cell subsets during pre-blip timepoint was calculated using CellChat v2 [88], following the standard workflow (from Anndata object) and keeping the default settings.

#### Gene set enrichment analysis and scanpy score.

Gene set enrichment analysis (GSEA) was performed on selected MSigDB v7.5.1 Hallmark and KEGG genesets using the fgsea package (available on Bioconductor v3.18) in R (v4.3.3) and visualised with the GOChord function in the GOplot package. Briefly, genes were ranked in the descending order by the Wilcoxon statistic value from the pairwise Wilcoxon rank sum test (via ‘tl.rank_genes_groups’ in scanpy, comparisons: Blip vs. pre-Blip, Post-blip vs. Pre-blip). Expression scores for selected MSigDB v7.5.1 Hallmark & KEGG and GOBP genesets (GO:0050864, GO:0030888, GO:0045577) were generated by using scanpy.tl.score_genes function.

#### Pseudotime analysis.

GEX-based pseudotime and trajectory inference was performed on the whole B cell dataset using slingshot package (available on Bioconductor v3.18) [89] in R (v4.3.3) with default settings but specifying start.clust = ‘Naive1’. VDJ-based pseudotime inference was performed using ‘VDJ pseudobulk feature space’ workflow as outlined within dandelion package v0.3.3 [90].

#### BCR pre-processing.

Single-cell V(D)J data from the 5′ Chromium 10x kit were initially processed with Cell Ranger multi pipeline (v7.0.1). BCR contigs contained in ‘filtered_contigs.fasta’ and ‘filtered_contig_annotations.csv’ from all samples were pre-processed using ‘immcantation’ inspired pre-processing pipeline [90] implemented in the dandelion package v0.3.3 via Singularity following the standard workflow and recommended parametrization. This Singularity-based pre-processing also incorporates mutational burden quantification via SHazaM’s observedMutations [66]. Dandelion output files from each sample were then concatenated into a vdj object using dandelion function concat(). Contigs assigned to cells that passed QC on the transcriptome data were retained for further QC assessment using dandelion function pp.check_contigs() [90].

#### B cell clone/clonotype analysis.

BCRs were grouped into clones/ clonotypes using dandelion package function dl.tl.find_clones(), which applies the following sequential criteria to both heavy- and light-chain contigs: (1) identical V and J gene usage, (2) identical junctional CDR3 amino acid length, and (3) at least 85% amino acid sequence similarity at the CDR3 junction (based on hamming distance). Light-chain pairing was performed using the same criteria within each heavy-chain clone [90]. BCR networks were subsequently constructed using dandelion function: tl.generate_network(), which is based on adjacency matrices computed from pairwise Levenshtein distance of the full amino acid sequence alignment for BCR(s) contained in every pair of cells within each timepoint. Construction of the Levenshtein distance matrices were performed separately for heavy-chain and light-chain contigs, and the sum of the total edit distance across all layers/matrices was used as the final adjacency matrix as described previously [90]. Weighted clonal overlap between each B cell subset at a single timepoint was calculated with dandelion function: tl.clone_overlap() with default settings [90]. Clonality was assessed by calculating clone size Gini index after running dandelion function: tl_clone_diversity() with metric = ”clone size”. This diversity metric does not rely on BCR network [90].

### Statistics

Neutralization and binding data were analysed using GraphPad Prism. For BCR phylogenetic network analysis statistical significance was assessed using pairwise Mann-Whitney U tests with the compare_means function from the ggpubr package (p > 0.05 not marked, p < 0.05 *, p < 0.01, **, p < 0.001 ***, p < 0.0001 ****). IgG subclass usage and nucleotide somatic hypermutation levels (V + J gene) of sequenced memory B cell populations were analysed using a one way-ANOVA with Tukey’s multiple comparison used for statistical testing. *p > 0.032, **p > 0.021, ***p > 0.0002 and ****p > 0.0001. Comparative analysis of differential gene expression from cell subsets used a Wilcoxon rank-sum test with Benjamini-Hochberg correction used for statistical testing, only genes with adjusted p-value < 0.05 and log-fold change >1 shown.

## Supporting information

S1 Fig Gating strategy for the assessment and sorting of memory B cell subsets. TLM (**A**), Resting (**B**) or Activated (**C**) memory B cells were sorted from the IgM+ and IgG+ populations.
(TIFF)

S2 Fig Env response during viral blip. **A** Anti-Env IgG plasma titre of the index and a HIV negative participant assessed against the heterologous B41 Env (Clade B) and BG505 N332 Env (Clade A). **B** Pseudovirus neutralization 50% inhibitory titres (ID50) of plasma from the index and a HIV negative participant against the heterologous B41 (clade B) and BG505 N332 (clade A) env sequences. MLV encoding pseudovirus was included as a control to confirm the absence of ART.
(TIFF)

S3 Fig Transient viral blip induces a complex response across both B- and non-B cells. **A-B** UMAP visualization of all single-cell transcriptomes (48,602 cells) as described in Fig 4A and annotated by sequencing sample **A** and timepoint **B**. **C** Comparison CellTypist automated annotation (left labels) and manually adjusted annotation (bottom labels) of clusters identified in Fig 4A, expressed as mean probability (circle colour). Circle size represents what fraction of manually annotated cluster was assigned to each CellTypist-annotated cluster. **D** UMAP visualization of all cells described in Fig 4A coloured by their normalized expression of CD19, CD4 and CD8. **E** Expression of top 10 marker genes for B and non-B cells annotated in Fig 4A. Wilcoxon rank-sum test with Benjamini-Hochberg correction used for statistical testing, only genes with adjusted p-value < 0.05 and log-fold change >1 shown. The fraction of cells in each group expressing an indicated gene is reflected by the dot size and the mean gene expression by the dot colour. **F-G** UMAP visualization of non-B cells selected from the dataset described in Fig 4A coloured by **F** cell subset annotation and **G** log-fold change (LFC) in their differential abundance across timepoints (generated by milopy package).
(TIF)

S4 Fig Transient viral blip results in transcriptomic and proteomic changes in B cell subsets. **A** Expression of top 10 marker genes for B cell subsets annotated in Fig 4E. Wilcoxon rank-sum test with Benjamini-Hochberg correction used for statistical testing, only genes with adjusted p-value < 0.05 and log-fold change >1 shown. The fraction of cells in each group expressing an indicated gene is reflected by the dot size and the mean gene expression by the dot colour. **B** UMAP visualization of B cells from Fig 4E, comparing expression of selected markers at RNA (GEX, top plots) and surface protein level (CITE-seq, bottom plots). **C-D** Heatmaps depicting number **C** and strength **D** of inferred cell-cell interactions at viral blip across all annotated cell clusters described in Fig 4A. **E** UMAP visualization of 42,149 B and plasma cells described in Fig 4A coloured by log-fold change (LFC) in their differential abundance across timepoints (generated by milopy package).
(TIF)

S5 Fig Variable region gene usage and mutation burden across B cell subsets and timepoints. **A** Heavy chain V gene usage by elite controller B cells shown in Fig 4A stratified by subset and timepoint. **B** Exemplary pre-blip mixed clones containing TLM2 cells selected from Fig 5A, coloured by mutation count (left) and subset annotation (right).
(TIF)
