# Supplementary figures and images for: Multifaceted B cell response to transient HIV viremia in elite controllers
Source: PLoS Pathog. 2026 Jan 16;22(1):e1013817. doi: 10.1371/journal.ppat.1013817 (PMC12890225; doi:10.1371/journal.ppat.1013817)

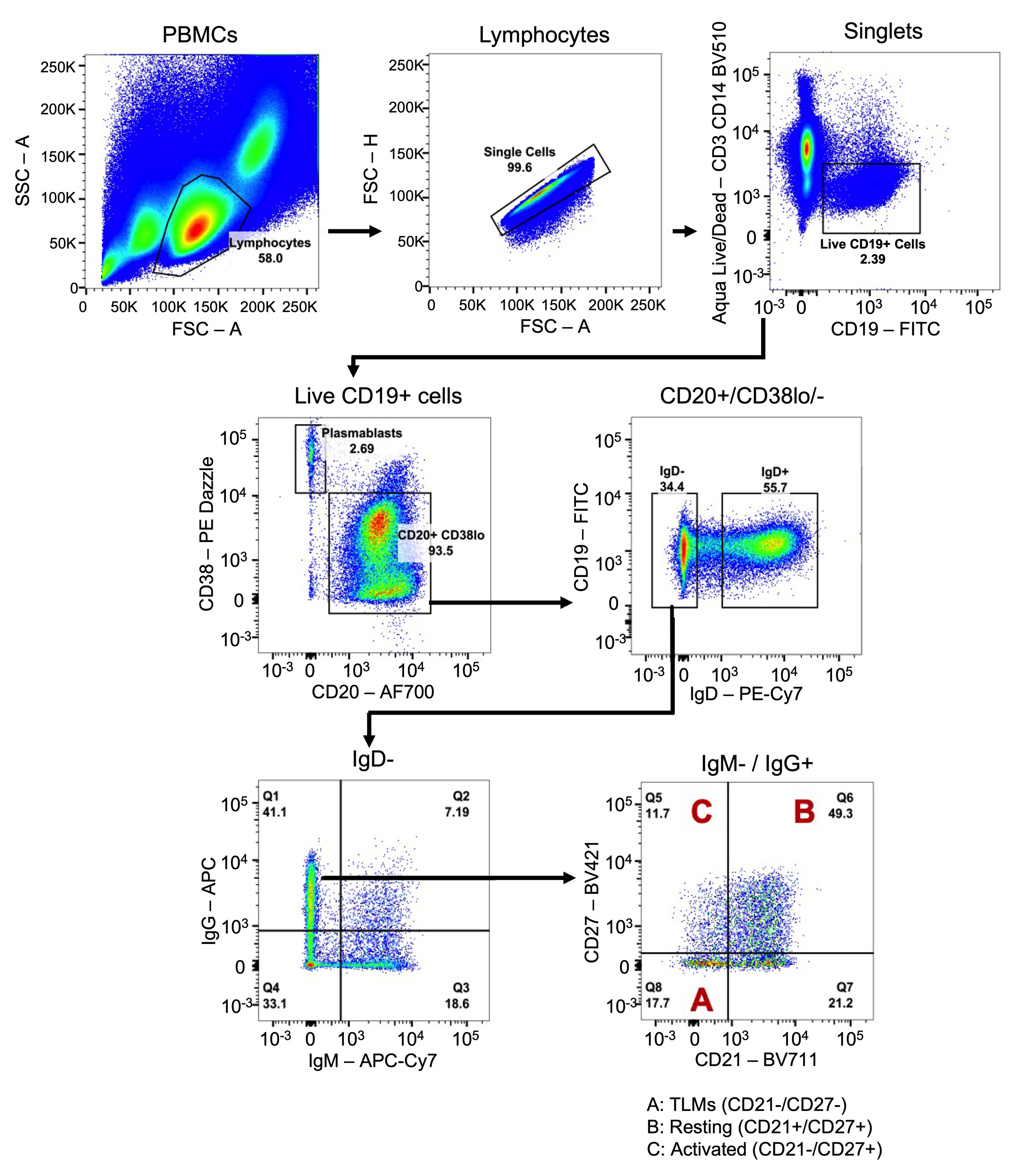

Supplement: S1 Fig — TLM (A), Resting (B) or Activated (C) memory B cells were sorted from the IgM+ and IgG+ populations. (TIFF) [file ppat.1013817.s001.tiff]

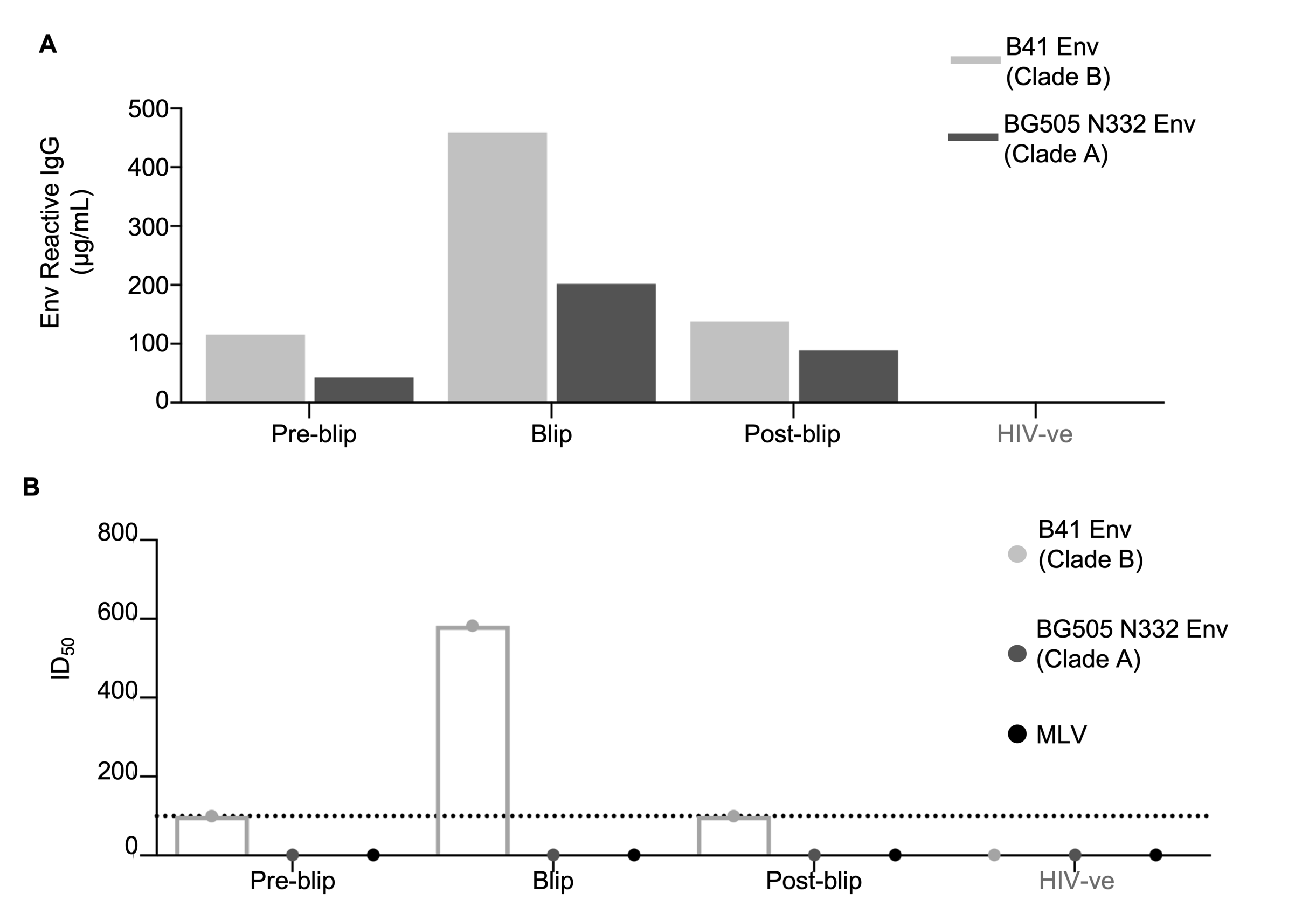

Supplement: S2 Fig — A Anti-Env IgG plasma titre of the index and a HIV negative participant assessed against the heterologous B41 Env (Clade B) and BG505 N332 Env (Clade A). B Pseudovirus neutralization 50% inhibitory titres (ID50) of plasma from the index and a HIV negative participant against the heterologous B41 (clade B) and BG505 N332 (clade A) env sequences. MLV encoding pseudovirus was included as a control to confirm the absence of ART. (TIFF) [file ppat.1013817.s002.tiff]

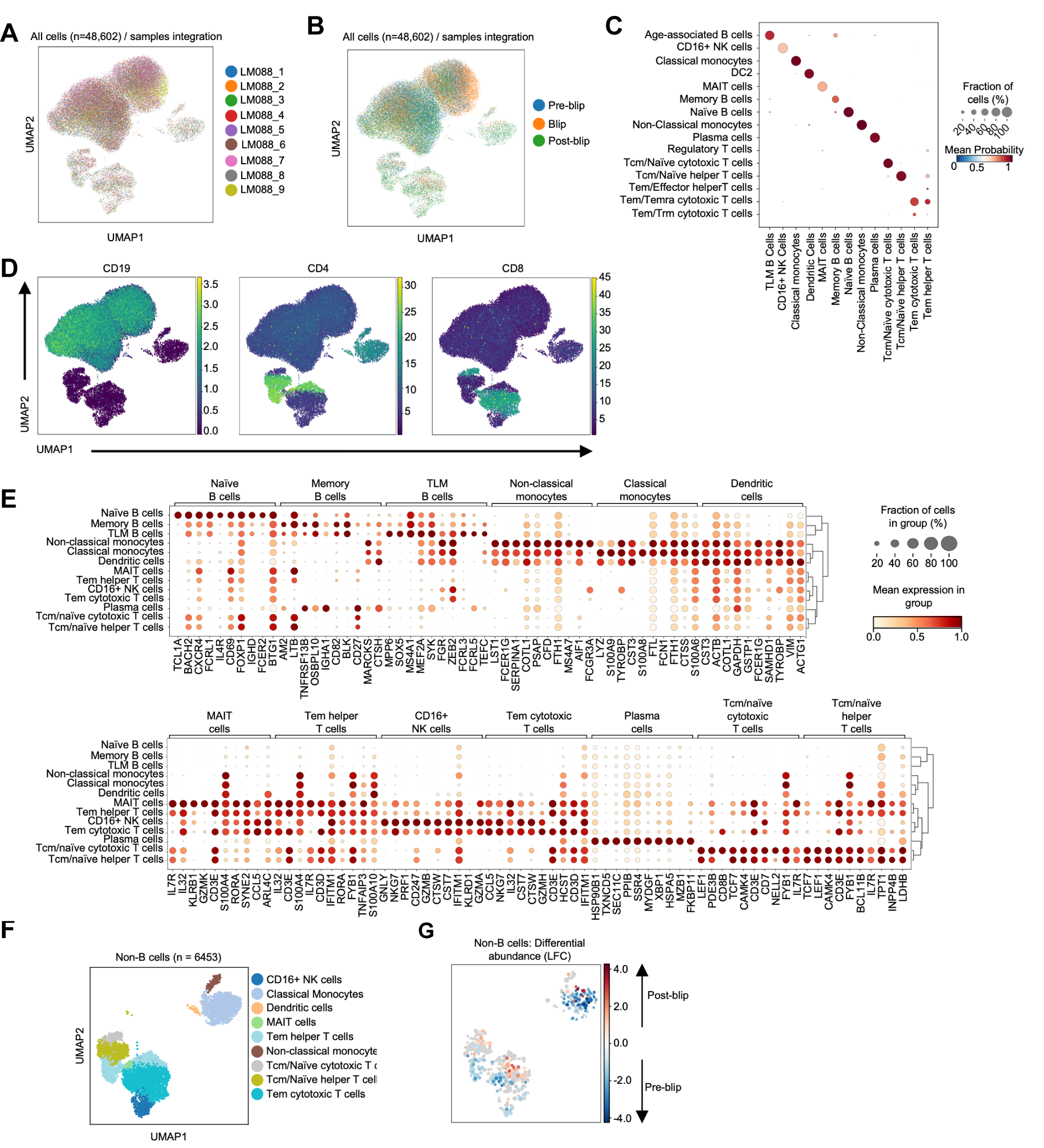

Supplement: S3 Fig — A-B UMAP visualization of all single-cell transcriptomes (48,602 cells) as described in Fig 4A and annotated by sequencing sample A and timepoint B. C Comparison CellTypist automated annotation (left labels) and manually adjusted annotation (bottom labels) of clusters identified in Fig 4A, expressed as mean probability (circle colour). Circle size represents what fraction of manually annotated cluster was assigned to each CellTypist-annotated cluster. D UMAP visualization of all cells described in Fig 4A coloured by their normalized expression of CD19, CD4 and CD8. E Expression of top 10 marker genes for B and non-B cells annotated in Fig 4A. Wilcoxon rank-sum test with Benjamini-Hochberg correction used for statistical testing, only genes with adjusted p-value < 0.05 and log-fold change >1 shown. The fraction of cells in each group expressing an indicated gene is reflected by the dot size and the mean gene expression by the dot colour. F-G UMAP visualization of non-B cells selected from the dataset described in Fig 4A coloured by F cell subset annotation and G log-fold change (LFC) in their differential abundance across timepoints (generated by milopy package). (TIF) [file ppat.1013817.s003.tif]

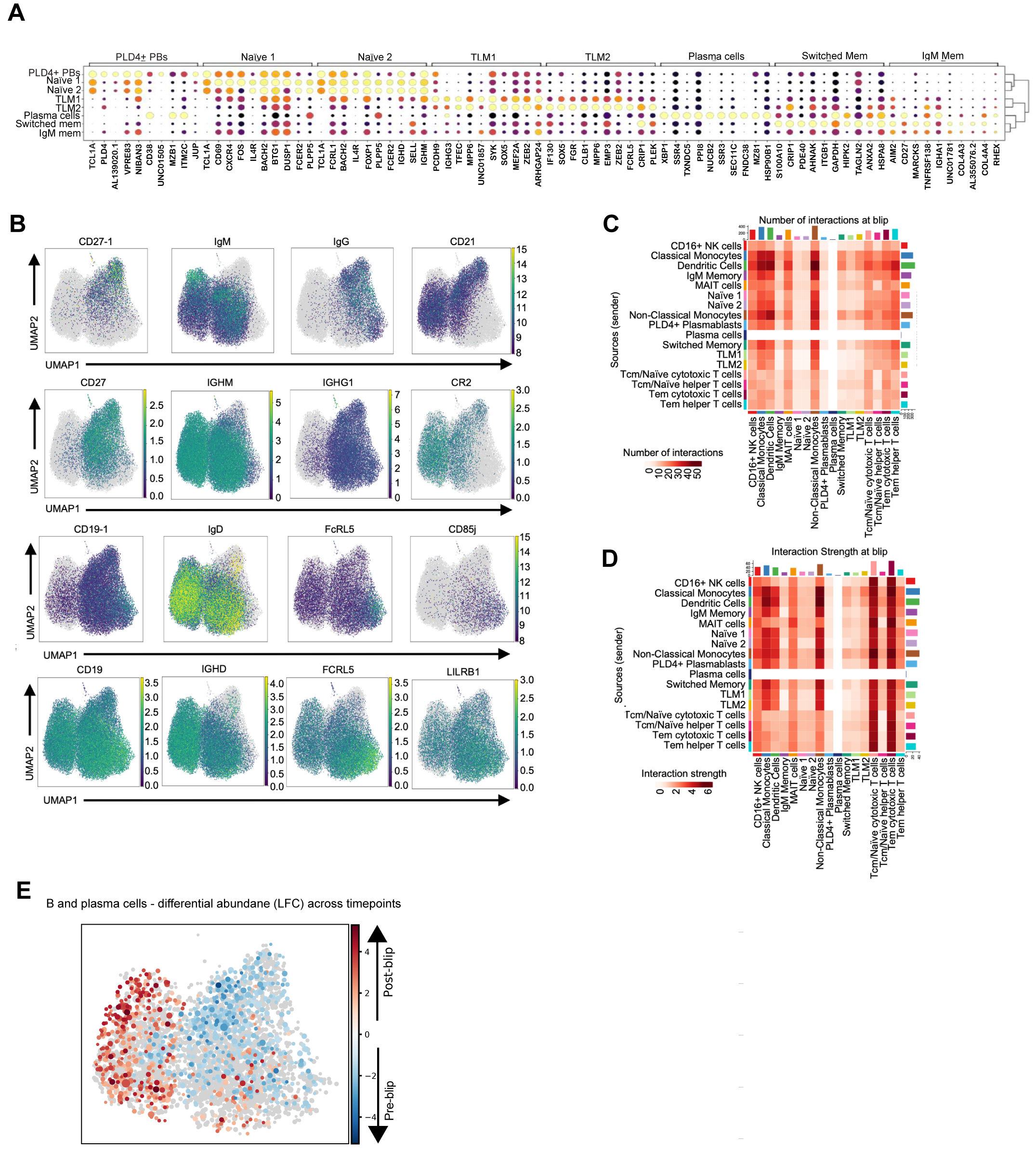

Supplement: S4 Fig — A Expression of top 10 marker genes for B cell subsets annotated in Fig 4E. Wilcoxon rank-sum test with Benjamini-Hochberg correction used for statistical testing, only genes with adjusted p-value < 0.05 and log-fold change >1 shown. The fraction of cells in each group expressing an indicated gene is reflected by the dot size and the mean gene expression by the dot colour. B UMAP visualization of B cells from Fig 4E, comparing expression of selected markers at RNA (GEX, top plots) and surface protein level (CITE-seq, bottom plots). C-D Heatmaps depicting number C and strength D of inferred cell-cell interactions at viral blip across all annotated cell clusters described in Fig 4A. E UMAP visualization of 42,149 B and plasma cells described in Fig 4A coloured by log-fold change (LFC) in their differential abundance across timepoints (generated by milopy package). (TIF) [file ppat.1013817.s004.tif]

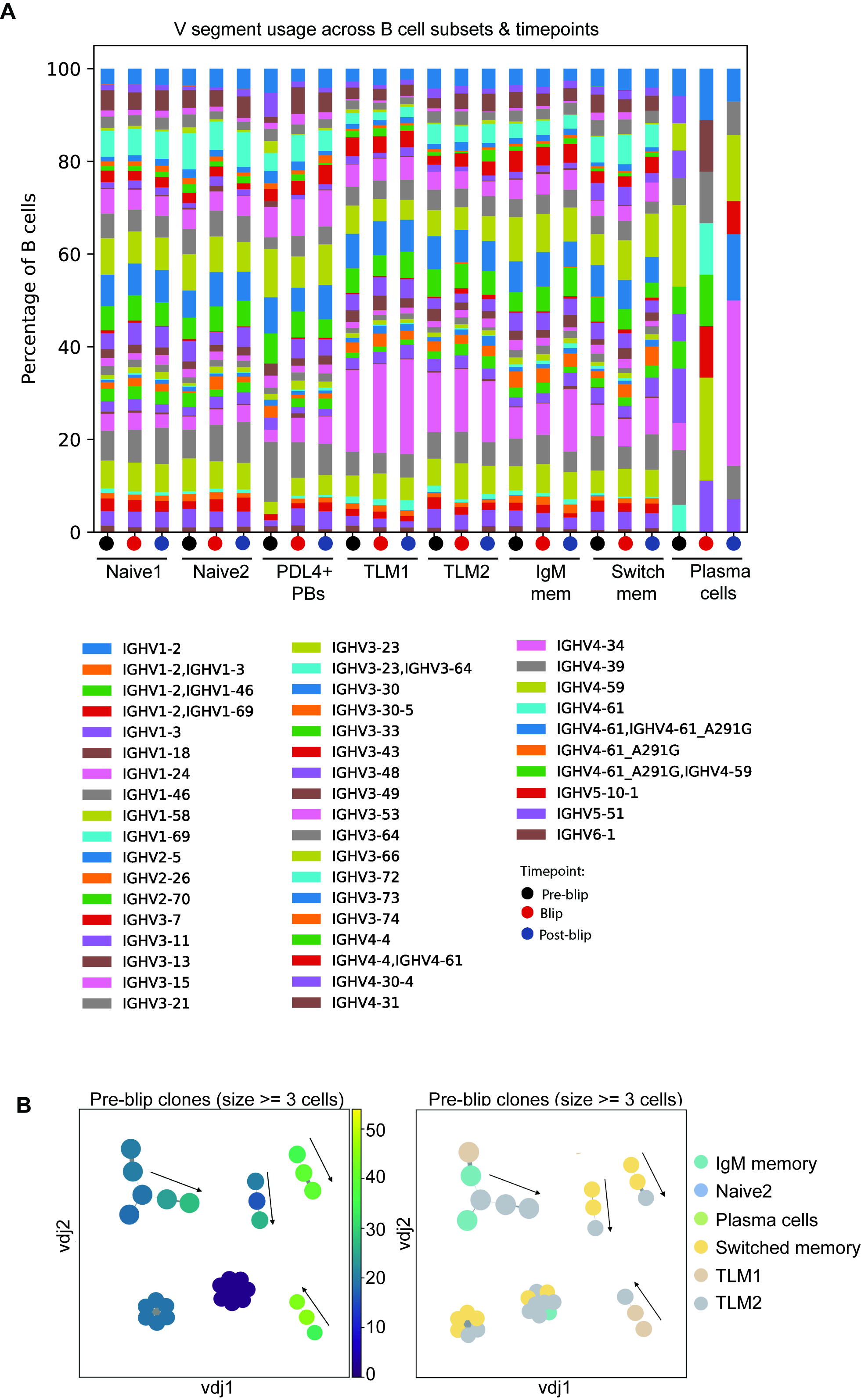

Supplement: S5 Fig — A Heavy chain V gene usage by elite controller B cells shown in Fig 4A stratified by subset and timepoint. B Exemplary pre-blip mixed clones containing TLM2 cells selected from Fig 5A, coloured by mutation count (left) and subset annotation (right). (TIF) [file ppat.1013817.s005.tif]
